# Potential for genomic instability associated with retrotranspositionally-*incompetent* L1 *loci*

**DOI:** 10.1093/nar/gku687

**Published:** 2014-08-20

**Authors:** Kristine J. Kines, Mark Sokolowski, Dawn L. deHaro, Claiborne M. Christian, Victoria P. Belancio

**Affiliations:** Department of Structural and Cellular Biology, Tulane School of Medicine, Tulane Cancer Center, and Tulane Center for Aging, New Orleans, LA 70112, USA

## Abstract

Expression of the L1 retrotransposon can damage the genome through insertional mutagenesis and the generation of DNA double-strand breaks (DSBs). The majority of L1 *loci* in the human genome are 5′-truncated and therefore incapable of retrotransposition. While thousands of full-length L1 *loci* remain, most are retrotranspositionally-*incompetent* due to inactivating mutations. However, mutations leading to premature stop codons within the L1 ORF2 sequence may yield truncated proteins that retain a functional endonuclease domain. We demonstrate that some truncated ORF2 proteins cause varying levels of toxicity and DNA damage when chronically overexpressed in mammalian cells. Furthermore, transfection of some ORF2 constructs containing premature stop codons supported low levels of Alu retrotransposition, demonstrating the potential for select retrotranspositionally-*incompetent* L1 *loci* to generate genomic instability. This result suggests yet another plausible explanation for the relative success of Alu elements in populating the human genome. Our data suggest that a subset of retrotranspositionally-*incompetent* L1s, previously considered to be harmless to genomic integrity, may have the potential to cause chronic DNA damage by introducing DSBs and mobilizing Alu. These results imply that the number of known L1 *loci* in the human genome that potentially threaten its stability may not be limited to the retrotranspositionally active *loci*.

## INTRODUCTION

Expression of the Long INterspersed Element-1 (LINE-1 or L1) retrotransposon can compromise genomic integrity through insertional mutagenesis, rearrangements generated by non-allelic homologous recombination and the generation of DNA double-strand breaks (DSBs) (1–6). Endogenous L1 expression has been detected in the germ line, as well as in multiple normal human tissues and adult stem cells (7–9). In addition, L1 expression is also significantly elevated in most human cancers when compared to matched normal tissues (10–14). These reports support the possibility that ongoing, low-level L1 activity can introduce structural genomic rearrangements over the life span of an organism. Such rearrangements could be mutagenic and potentially contribute to tumor initiation and/or progression.

L1 causes insertional mutagenesis through self-retrotransposition as well as through the mobilization of non-autonomous retrotransposons, such as Alu and SVA elements, which rely on the L1-encoded ORF2 protein for their propagation (15,16). Indeed, recent next-generation sequence analyses have confirmed that several types of human tumors harbor *de novo* L1 insertions, some of which have landed in genes relevant to tumorigenesis (13,14). Once integrated into the genome, copies of L1 and L1-driven retroelements provide abundant substrates for non-allelic homologous recombination events, which have been reported to result in deletions, duplications, translocations and other genomic rearrangements (4,17–21). Collectively, these types of L1-induced alterations of the genome have resulted in a variety of diseases, including multiple cancers [reviewed in (22)].

In addition to retrotransposition, L1 expression has been reported to generate DSBs in both normal and cancer cells (6,9,23). Both L1-driven retrotransposition events and L1-induced DSBs depend on the endonuclease activity of the L1 ORF2 protein, which initiates the integration process by nicking the host DNA (24). Although the origin of the second-strand nick required for completion of the retrotransposition process is unknown, it has been established that expression of the L1 ORF2 protein containing a functional endonuclease domain results in the formation of DSBs (6,9,23). Importantly, it is estimated that L1-induced DSBs are much more frequent than successful L1 retrotransposition events under the same transfection conditions in HeLa cells (6). While the specific consequences from L1-induced DSBs are not yet fully known, DSBs generally are highly mutagenic in mammalian cells, and are known to contribute to genomic instability and cancer progression [reviewed in (25–27)]. Consistent with this premise, it was reported that L1-induced DSBs may contribute to translocations in prostate cancer cells, suggesting that L1 activity may be involved in prostate cancer progression (28).

The cellular response to the genomic damage generated by expression of the functional L1 ORF2 protein can cause toxicity or cell cycle arrest, leading to a decrease in cellular viability or a reduction in cellular proliferation (9,29). Several reports have demonstrated that L1 toxicity manifests through the induction of apoptosis (6,23,29,30) or a senescence-like state in both normal and cancer cells (9,29).

**Figure 1.**
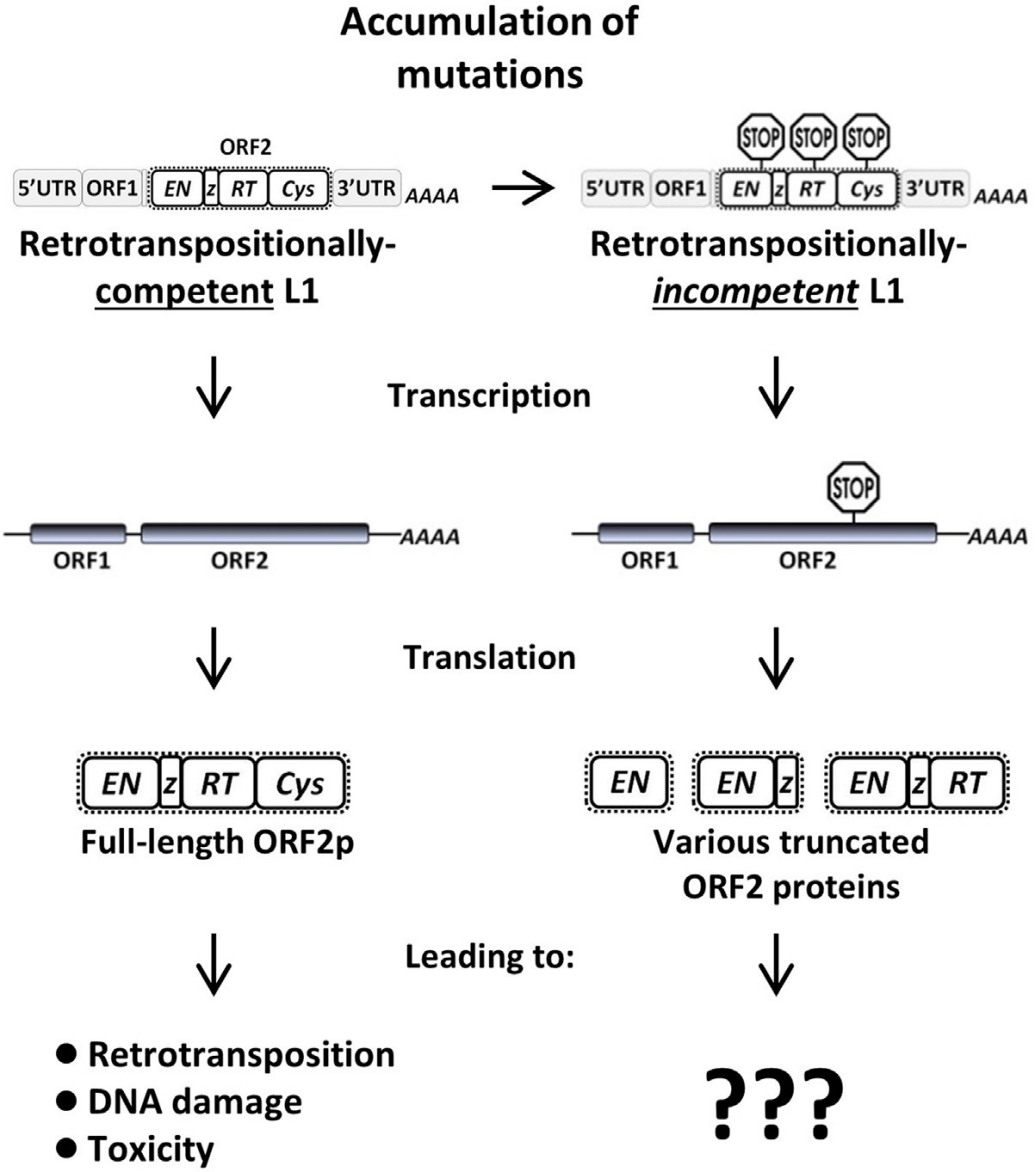
Consequences of the expression of retrotranspositionally-*incompetent* L1 *loci* are unknown. Retrotranspositionally-competent L1 *loci* accumulate mutations over time, eventually rendering them unable to further retrotranspose. These now retrotranspositionally-*incompetent* L1 *loci* may still be actively expressed. Truncated L1 proteins may potentially be expressed from mutated *loci* that have acquired premature stop codons. It is unknown if such truncated proteins are stable and functional, or capable of causing genomic instability.

L1 encodes a bicistronic messenger RNA (mRNA) that produces two proteins, ORF1 and ORF2, which are both required for retrotransposition (31). Even though there are over 500 000 L1 copies in the human genome, the vast majority are retrotranspositionally-*incompetent* due to 5′ truncations or inactivating mutations within the ORF1 or ORF2 sequences (32–34). However, some of these retrotranspositionally-*incompetent* L1 *loci* are still expressed (35), and the consequences of their expression have yet to be explored. Full-length, retrotranspositionally-*incompetent* L1 *loci* containing premature stop codons within the ORF2 sequence are of particular interest, because although their retrotransposition is precluded, such mutated *loci* could potentially produce truncated ORF2 proteins with a functional endonuclease domain (Figure 1). It is unknown whether such truncated L1 ORF2 proteins would be stable and functional in mammalian cells, but *in vitro* studies of ORF2 domains provide a precedent that supports this possibility (24,36).

The endonuclease domain of the L1 ORF2 protein has been successfully expressed *in vitro*, with the truncated protein retaining nicking activity and cleavage site preference (24). These *in vitro* studies suggested that the nicking activity of the endonuclease is actually repressed in the context of the full-length L1 ORF2 protein when compared to that of the endonuclease domain expressed independently (36). Expression of truncated ORF2 proteins from L1 *loci* containing premature stop codons could be an additional source of genomic instability if the endonuclease domain remains functional in a mammalian cellular environment.

In this study, we investigated the potential for L1 to produce stable, truncated ORF2 proteins capable of causing DNA damage in cultured mammalian cells. Our data demonstrate that truncated ORF2 proteins retain various levels of stability when expressed in mammalian cells, and that some truncated ORF2 proteins containing an intact endonuclease domain can induce cellular toxicity and DNA damage. Furthermore, our results show that transient transfection of some L1 and ORF2 expression plasmids containing a premature stop codon can drive Alu retrotransposition. Our findings suggest the possibility that some retrotranspositionally-*incompetent* L1 *loci*, previously considered to be inactive and innocuous, may be capable of inducing low levels of chronic genomic instability through the generation of DSBs and Alu mobilization.

## MATERIALS AND METHODS

### Bioinformatic analysis

L1Base (37) was searched to identify full-length L1 *loci* in the human and mouse genomes that are retrotranspositionally-*incompetent*, but still have the potential to express truncated L1 ORF2 proteins. The search criteria were chosen to identify L1 *loci* that contain an intact ORF1 sequence (no gaps, premature stops or frame shifts) and a mostly intact ORF2 sequence (no gaps or frame shifts) with at least one premature stop codon. Ninety-two human and 100 mouse *loci* conforming to these parameters were further analyzed to locate the position of the first stop codon within ORF2. Human L1.3 and mouse L1spa ORF2 were used as reference sequences (38,39).

### RT-polymerase chain reaction analysis

Total RNA was extracted from HeLa cells and poly(A) selected as previously described (40). Complementary DNA (cDNA) was synthesized from the poly(A) selected mRNA using the Reverse Transcription System kit (Promega) according to the manufacturer's protocol. As a negative control, a parallel reaction was included using mRNA without reverse transcriptase to detect DNA contamination. Primers were designed to target the endonuclease region (amino acids 1–239) of human L1.3 as follows: 5′-ATGACAGGATCAAATTCACACATAACA and 5′-AATCCTGAGTTCTAGTTTGATTGCACT. Polymerase chain reaction (PCR) was performed using 5 μl of HeLa cDNA, GoTaq Hot Start mastermix reagents (Promega) and 25 cycles of 94°C for 30 s, 57°C for 30 s and 72°C for 45 s. PCR products were cloned into pSC-A-amp/kan using the StrataClone PCR Cloning kit (Agilent) per the manufacturer's protocol and subsequently sequenced. Sequences were analyzed and mapped to the human genome (GRCh38 assembly) using the BLAT genome search (41) and the UCSC Genes and RefSeq Genes gene annotation tracks. Due to its potential to produce a truncated ORF2 protein, the L1 *locus* highlighted in Supplementary Table S1 was further analyzed using the following additional gene annotation tracks: GENCODE Genes V19, Genscan Gene, MGC Genes, ORFeome Clones, Pfam in UCSC Gene and UCSC Alt Events. The GENCODE Genes V19 track was the only one that placed the location of this L1 *locus* within an overlapping transcript. This transcript appears to be specific to interferon (IFN)-treated K562 cells and it was not identified in any other cell lines tested in the study, including untreated K562 cells (42,43). Based on these findings, this L1 *locus* is annotated as being located in an intergenic region in Supplementary Table S1.

### Plasmids

#### EN(+), EN(+)z, EN(+)RT and mEN(+)

Previously reported plasmids containing wild-type human L1 ORF2 (9), codon-optimized human L1 (29,44) and codon-optimized mouse L1 ORF2 sequences (45) were utilized as templates to construct expression plasmids for the truncated ORF2 proteins. We used PCR amplification to add a 5′-NheI restriction site and a premature stop codon (TGA) and 3′-BamHI restriction site to the 5′ and 3′ ends of the open reading frames, respectively. Primer sequences are available upon request. These PCR products were subsequently digested with the enzymes listed above and cloned into the similarly digested mammalian expression vector, pcDNA3.1/Hygro+ (Invitrogen). The EN expression vector encodes amino acids 1–239 from the human L1 ORF2; ENz encodes amino acids 1–490; and ENRT encodes amino acids 1–773 (Figure 3A). The mEN expression vector encodes amino acids 1–258 from the mouse L1 ORF2.

**Figure 2.**
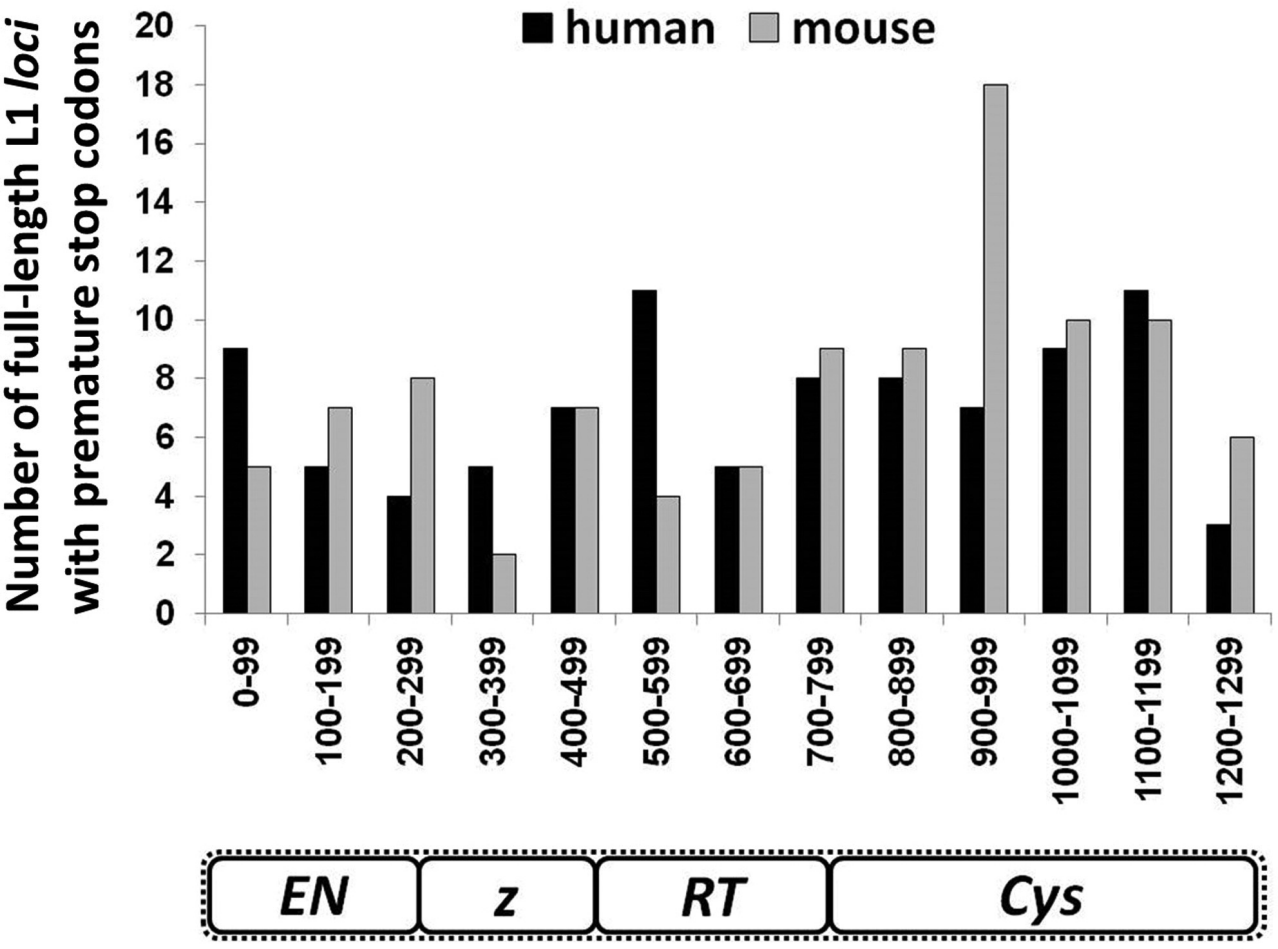
Identification of full-length retrotranspositionally-*incompetent* L1 *loci* containing premature stop codons in the human and mouse genomes. Bioinformatic analysis using L1Base (37) revealed numerous full-length retrotranspositionally-*incompetent* L1 *loci* containing an intact ORF1 (no gaps, premature stops or frame shifts) and an ORF2 (no gaps or frame shifts) with at least one stop codon. The histogram depicts the location of the first premature stop codon noted within the ORF2 amino acid sequence for human (black) and mouse (gray) L1 *loci* fitting these criteria (*X*-axis), with the number of such *loci* represented on the *Y*-axis.

**Figure 3.**
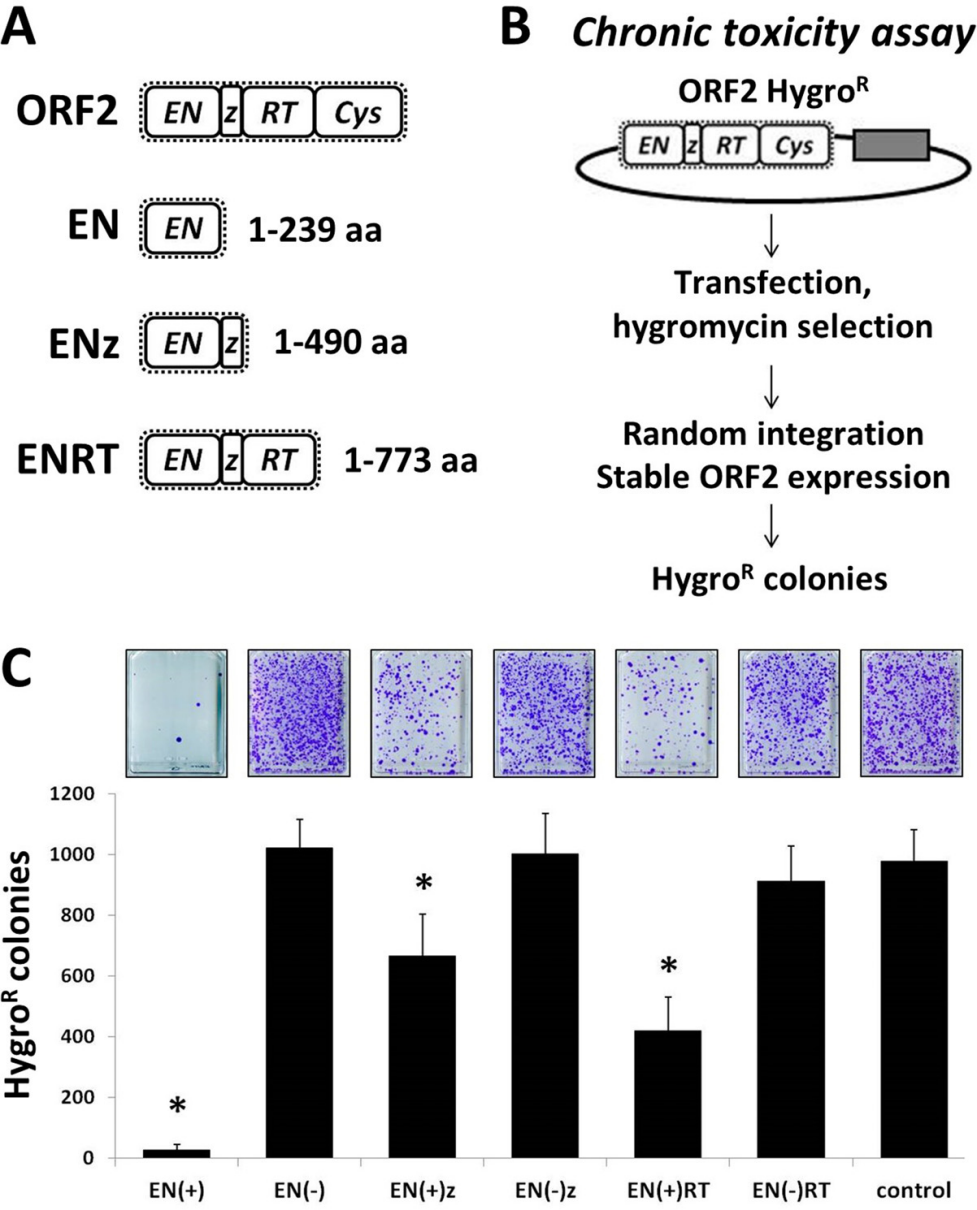
Stable transfection of various truncated ORF2 expression plasmids causes toxicity in human cells. (**A**) Schematic representation of truncated ORF2 constructs. Figures depict human L1 ORF2 domains and the amino acid position of the truncation. Endonuclease (EN), z-motif (z), reverse transcriptase (RT) and Cys-domain (Cys). (**B**) Experimental approach for the chronic toxicity assay: cells are transfected with a plasmid containing both Hyg^R^ and the truncated ORF2 gene. Selection with hygromycin allows for the stable selection of the cells harboring the ORF2 expression plasmids. Colony formation can be used as a measure of relative toxicity by comparing the number of Hyg^R^ colonies that survived after stable expression of the functional and non-functional truncated ORF2 proteins (29). (**C**) Results from four independent chronic toxicity assays indicate a significant reduction in the number of Hyg^R^ colonies (*Y*-axis) formed in 293 cells after stable expression of the indicated truncated human ORF2 construct (*X*-axis). Constructs are annotated as having functional endonuclease domains (+) or non-functional mutant endonuclease domains (−). Control indicates cells transfected with an empty vector. Asterisks indicate a statistically significant difference between each truncated ORF2 expression plasmid containing a functional endonuclease and its non-functional counterpart (*t*-test, *P* ≤0.01).

#### EN(−), EN(−)z and EN(−)RT

The codon-optimized truncated ORF2 endonuclease mutant (non-functional) constructs contain two previously characterized mutations in the endonuclease domain of ORF2; D205A and H230A (24,46). Truncated wild-type ORF2 endonuclease mutant expression plasmids contain the D205A mutation, introduced by the QuikChange Site-Directed Mutagenesis kit (Stratagene) per the manufacturer's protocol using the following primers: 5′-CCACACCTATTCCAAAATTGCCCACATAGTTGGAAGTAAAG and 5′-CTTTACTTCCAACTATGTGGGCAATTTTGGAATAGGTGTGG (46).

To generate the ENz and ENRT nuclear localization signal (NLS) mutant plasmids, we used cNLS mapper to identify a putative NLS within the z-motif region of ORF2 (amino acids 382–385). We used site-directed mutagenesis to introduce mutations into this location (47). The following primers were used to change amino acids 382–385 from KKKR→AAAA: 5′-CCCTGGCCCGGCTGATCGCGGCGGCGGCGGAGAAGAACCAGATCGAC and 5′-GTCGATCTGGTTCTTCTCCGCCGCCGCCGCGATCAGCCGGGCCAGGG.

#### L1 and ORF2 EN*rtcys and ENRT*cys

We introduced premature stop codons into the codon-optimized, full-length ORF2 expression vector (pBudCE4.1; Invitrogen) previously reported in Wallace *et al*., 2008 (29) using site-directed mutagenesis. Premature stop codons were inserted after amino acid position 239 (K240* or AAG→TAG) to create EN*rtcys, or after position 773 (T774* or ACT→TGA) to create ENRT*cys using the following primer sets: 5′-CTGGAGCTGCGGATCTAGAACCTGACCCAGAG, 5′-CTCTGGGTCAGGTTCTAGATCCGCAGCTCCAG; and 5′-CTGGGCATCCAGCTGTGACGGGACGTCAAGGACCTG, 5′-CAGGTCCTTGACGTCCCGTCACAGCTGGATGCCCAG. Premature stop codons were introduced into the codon-optimized, full-length L1 expression vector (pBlueScript II; Stratagene) previously reported in Wagstaff *et al*. (44), into the same locations within ORF2 as listed above. Endonuclease mutant versions of the premature stop codon constructs were generated by using site-directed mutagenesis to change amino acid 205 from Asp to Ala using the following primers: 5′-CCTACAGCAAGATCGCCCACATCGTGGGCAG and 5′-CTGCCCACGATGTGGGCGATCTTGCTGTAGG (46).

The pIRES2-GFP expression plasmid employed in the acute toxicity assay to confer G418 resistance has been described previously (6). The Neo^R^-tagged Alu retrotransposition reporter plasmid used in the retrotransposition assay has been described previously (15).

### Cell culture and transfections

293-FRT (Invitrogen) cells were cultured in Dulbecco's modified Eagle's medium (Hyclone) supplemented with 10% fetal bovine serum and maintained at 37°C in 6% CO_2_. HeLa and NIH-3T3 cells were maintained as previously described (40). For all experiments, cells were seeded 16–18 h prior to transfection, and normal growth media was replaced 3 h post-transfection.

#### Chronic toxicity assays

293-FRT cells were seeded at a density of 125 000 cells per T75 flask and transfected with 400 ng of the truncated ORF2 expression plasmids, using 12 μl of Lipofectamine (Invitrogen) and 4 μl of Plus.

#### Acute toxicity assays

293-FRT cells were seeded at a density of 125 000 cells per T75 flask and co-transfected the following day with 100 ng of the Neo^R^ expression plasmid, and either 400 ng of the truncated ORF2 expression plasmids or 800 ng of the premature stop ORF2 expression plasmids, using 12 μl of Lipofectamine and 4 μl of Plus.

#### Western blot

To analyze total protein expression, 1.3–2 million 293-FRT cells were seeded per T25 flask and transfected the next day with 0.125–3 μg of the various ORF2 expression vectors, using 8 μl of Lipofectamine and 4 μl of Plus. NIH-3T3 cells were seeded at a density of 250 000 cells per T25 flask and transfected the following day with 0.5–4 μg of plasmid, with 8 μl of Lipofectamine and 4 μl of Plus. For nuclear and cytoplasmic fractionation experiments, 4.5 million 293-FRT or 2 million HeLa cells were seeded per T75 flask and transfected with 6 μg of plasmid, using 25 μl of Lipofectamine and 12 μl of Plus. Cells were harvested for immunoblot analysis ∼24 h later, or 3–12 h post-transfection as specified in the time course experiment.

#### COMET assays

HeLa cells were seeded at a density of 750 000 cells per T25 and transfected the following day with 2 μg of the EN constructs, using 8 μl of Lipofectamine and 4 μl of Plus. Cells were harvested 24 h post-transfection for neutral COMET analysis.

#### Alu retrotransposition assay

Approximately 500 000 HeLa cells were seeded per T75 flask and co-transfected the following day with 500 ng of the Alu retrotransposition reporter plasmid and 400 ng of the L1 or ORF2 premature stop expression vectors, using 8 μl of Lipofectamine and 4 μl of Plus.

### Toxicity assay

Toxicity assay experiments were performed as previously described, with minor modifications (6,29).

#### Chronic toxicity assays

Truncated ORF2 sequences were cloned into the pcDNA3.1/Hygro+ mammalian expression vector, which also expresses a gene for hygromycin resistance (Hyg^R^). 293-FRT cells were transfected with the truncated ORF2 plasmids. Hygromycin selection (90 μg/ml) was started 48 h post-transfection and maintained for 2 weeks, allowing for the stable and constant expression of truncated ORF2 proteins throughout the entirety of the assay. Colonies were fixed and stained with a crystal violet solution (0.2% crystal violet, 5% acetic acid, 2.5% isopropanol).

#### Acute toxicity assays

293-FRT cells were transiently co-transfected with the pIRES2-GFP plasmid, which confers G418 resistance (Neo^R^) and either the truncated ORF2 or the premature stop ORF2 expression plasmids. Selection medium (500 μg/ml G418) was added the following day and maintained for 14 days to select for G418-resistant colonies. Colonies were fixed and stained as described above. For both the acute and chronic toxicity assays, transfections were performed in duplicate, and the experiments were repeated four times. Statistical significance was evaluated using Student's *t*-test for samples of equal variance. Error bars in figures represent standard deviations.

### Immunoblot analysis

Protein harvest and western blot analyses were conducted as previously described (48).

#### Total protein harvest

Cells were washed once with 1X phosphate buffered saline (PBS) and lysed in 300 μl TLB-sodium dodecyl sulphate (SDS) buffer [50 mM Tris, 150 mM NaCl, 10 mM ethylenediaminetetraacetic acid (EDTA), 0.5% SDS, 0.5% Triton-X, pH 7.2] supplemented with 10 μl/ml each of the Halt protease inhibitor cocktail (Pierce) and Phosphatase inhibitor cocktails 2 and 3 (Sigma). Harvested cells were sonicated three times for 10 s each at 12 W using a Microson XL-2000 sonicator (Misonix). Cell lysates were collected after centrifugation at 14 000 rpm for 15 min at 4°C.

#### Nuclear and cytoplasmic fraction separation

Cells were washed with 1X PBS and harvested in 500 μl TLB buffer (50 mM Tris, 150 mM NaCl, 10 mM EDTA, 0.5% Triton-X, pH 7.2) supplemented with the same protease and phosphatase inhibitor cocktails listed above. Nuclei were separated by centrifugation at 14 000 rpm for 15 min at 4°C, and the supernatant containing the cytoplasmic portion was collected. The nuclear pellet was resuspended in 200 μl TLB-SDS buffer supplemented with the same protease and phosphatase inhibitor cocktails as listed above. Samples were sonicated as described above, and the nuclear lysates were collected after centrifugation at 14 000 rpm for 15 min at 4°C.

#### Western blot

Protein concentration was calculated using the Bio-Rad Protein Assay for all lysates. Samples (35–45 μg) were boiled in denaturing Laemmli buffer supplemented with β-mercaptoethanol and fractionated on NuPAGE 4–12% Bis-Tris gels (Invitrogen). Proteins were transferred onto nitrocellulose membranes using the iBlot system (Invitrogen). Membranes were rinsed with PBS-Tween (1x PBS, 0.1% Tween), blocked with 5% non-fat dry milk in PBS-Tween and incubated with primary antibodies overnight at 4°C. ORF2 polyclonal antibodies were generated against amino acids 48–63 and 152–166 of the human L1 ORF2 endonuclease domain, as previously reported (7). Custom polyclonal rabbit antibodies were generated against amino acids 159–172 of the mouse L1 ORF2 endonuclease domain. Antibodies were all diluted in 3% milk in PBS-Tween as follows: human L1 endonuclease 1:500, mouse L1 endonuclease 1:500; GAPDH (Santa Cruz; sc-25778) 1:7000; γH2AX (Santa Cruz; sc-101696) 1:50 000–1:100 000; and Lamin A/C (Cell Signaling; 2032S) 1:1000. GAPDH was used to confirm equal loading of the gel, and also to demonstrate proper subcellular fractionation. γH2AX was used as an indicator of DNA damage, and Lamin A/C was used to verify satisfactory subcellular fractionation. Membranes were washed and incubated with the secondary antibody, either HRP-donkey anti-goat (Santa Cruz; sc-2020) or HRP-donkey anti-rabbit (Santa Cruz; sc-2317), at a 1:5000 dilution in 3% milk in PBS-Tween. Western blots were developed using the Immun-Star WesternC kit (Bio-Rad) or ECL Prime Western blotting detection reagents (Amersham), and the images were captured using a Bio-Rad Gel Doc XR+ imager. We used the Image Lab 4.0.1 software to quantify the signal intensity of observed bands. Transfections and subsequent immunoblot analyses were repeated a minimum of three times. Statistical significance was evaluated using Student's *t*-test for samples of equal variance. Error bars in figures represent standard deviations.

### COMET assay

The neutral COMET assay was completed as previously described (6,49) using the CometAssay kit (Trevigen), per the manufacturer's protocol. Briefly, HeLa cells were transiently transfected with the truncated EN expression plasmids and harvested 24 h post-transfection. Cells were resuspended in cold PBS and then mounted on an agarose-coated slide and treated with a lysis solution to remove the cellular and nuclear membranes. Samples were subsequently incubated in an alkali solution to unwind and denature the DNA, and subjected to gel electrophoresis. Cells were stained with SYBR Green and visualized using epifluorescence microscopy. Individual cells with DNA damage, as indicated by the presence of fragmented DNA, were scored against the total number of cells analyzed. COMET assays were repeated in triplicate and the statistical significance was evaluated using Student's *t*-test for samples of equal variance. Error bars in figures represent standard deviations.

### Alu retrotransposition assay

Alu retrotransposition experiments were performed as previously described (9). In brief, HeLa cells were co-transfected with the L1 or ORF2 premature stop expression vectors and the Neo^R^-tagged Alu retrotransposition reporter plasmid according to the transfection conditions described above (15). Selection medium (400 μg/ml G418) was started 24 h post-transfection and maintained for 14 days to select for G418-resistant colonies representing Alu retrotransposition events. Colonies were fixed and stained using a crystal violet solution as listed above. Alu retrotransposition assays were repeated in triplicate and the statistical significance was evaluated using Student's *t*-test for samples of equal variance. Error bars in figures represent standard deviations.

## RESULTS

### Human and mouse genomes contain retrotranspositionally-*incompetent* L1 *loci* that may potentially express truncated ORF2 proteins

Previous reports have estimated that there are ∼6000 full-length L1 *loci* per human genome, of which only 80–100 are retrotranspositionally-competent (32,37). However, the inability to retrotranspose does not exclude the possibility that some of the defective full-length L1 *loci* can still pose a threat to genomic stability, particularly if they can express truncated ORF2 proteins containing a functional endonuclease domain. To test this hypothesis, we identified full-length retrotranspositionally-*incompetent* L1 *loci* in the human and mouse genomes that could potentially express truncated L1 ORF2 proteins. We performed a bioinformatic analysis of human and mouse L1 *loci* using L1Base, a database of the full-length L1s present in human and rodent genomes (37). The search parameters were adjusted to only include full-length L1 *loci* encoding an intact ORF1 sequence (no gaps, premature stop codons or frame shifts), because it has been previously reported that the introduction of a premature stop codon within ORF1 reduces ORF2 protein expression from its bicistronic L1 transcript (50,51). These resulting matches were further narrowed by searching for L1 *loci* with an intact ORF2 sequence (no gaps or frame shifts) containing premature translation termination codons, which if expressed, may generate truncated ORF2 proteins. We identified 92 such full-length L1 *loci* in the human genome, and over 500 *loci* in the mouse genome. The position of the first stop codon present within the ORF2 coding sequence was registered for the entire collection of human L1 *loci* conforming to our search criteria, and for 100 randomly selected mouse L1 *loci* fitting these parameters (Figure 2). Using an RT-PCR approach, we also recovered endogenous L1 transcripts produced in HeLa cells and mapped their genomic location. This analysis identified 28 unique full-length L1 *loci*, all of which were retrotranspositionally-*incompetent*. Among these, we detected one L1 *locus* with the potential to produce an ORF2 protein truncated at amino acid 377 (Supplementary Figure S1, Supplementary Table S1). This particular L1 *locus* is located between the DMGDH (NM_013391) and BHMT (NM_001713) genes on chromosome 5, according to the UCSC Genes, RefSeq Genes, Genscan Gene, MGC Genes, ORFeome Clones, Pfam in UCSC Gene and UCSC Alt Events gene annotation tracks, and placed in the alternative DMGDH non-coding transcript by the ENCODE/GENCODE Genes V19 track. This non-coding genomic transcript appears to be specific to IFN-treated K562 cells and it was not identified in any other cell lines tested in the ENCODE study, including untreated K562 cells (42,43).

### Transfection of truncated L1 ORF2 constructs containing a functional endonuclease domain leads to toxicity

We generated plasmids containing codon-optimized human L1 ORF2 sequences designed to express truncated ORF2 proteins with either a functional or non-functional endonuclease domain [annotated as EN(+) and EN(−), respectively]. The C-terminal ends of the truncated ORF2 constructs were chosen based on the reported boundaries of the domains identified within the L1 ORF2 protein (Figure 3A), with EN representing the endonuclease domain alone (24), ENz including the endonuclease domain and z-motif (52), and ENRT encompassing the endonuclease, z-motif, and reverse transcriptase domains (53). Additionally, we created a codon-optimized construct for expression of the mouse L1 ORF2 endonuclease domain (mEN) (Supplementary Figure S3).

It has been previously reported that expression of L1 is toxic and that the toxicity is largely dependent upon the endonuclease activity of the ORF2 protein (6,29). We measured chronic and acute toxicity following the transfection of the above constructs using previously described colony formation assays (Figure 3B and Supplementary Figure S2) (6,29). Both stable and transient transfections of the EN(+), EN(+)z and EN(+)RT plasmids in 293 cells resulted in a significant amount of cellular toxicity as demonstrated by a reduction in the formation of Hyg^R^ and Neo^R^ colonies, respectively, in comparison to the toxicity observed with their corresponding non-functional counterparts, EN(−), EN(−)z and EN(−)RT (Figure 3C and Supplementary Figure S2). Transfection of the EN(+) expression plasmid caused a striking amount of both chronic and acute toxicity, reducing the number of Hyg^R^ and Neo^R^ colonies by over 95% when compared to its non-functional EN(−) counterpart or to the empty control. Transfection of the mEN(+) endonuclease construct in human 293 cells was also highly toxic in comparison to the control in both chronic and acute toxicity assays (Supplementary Figure S3). Although stable transfection of the EN(+)z and EN(+)RT constructs was not as toxic as that observed with the EN(+) construct, it nevertheless resulted in a significant decrease in the number of Hyg^R^ colonies (∼35 and 55%, respectively), when compared to results obtained with their endonuclease-mutant counterparts (Figure 3C). As expected, transient transfection of these constructs resulted in less toxicity compared to stable transfection, causing a 25 and 35% reduction in the number of G418^R^ colonies, respectively (Supplementary Figure S2).

### Expression of truncated L1 ORF2 proteins in mammalian cells

**Figure 4.**
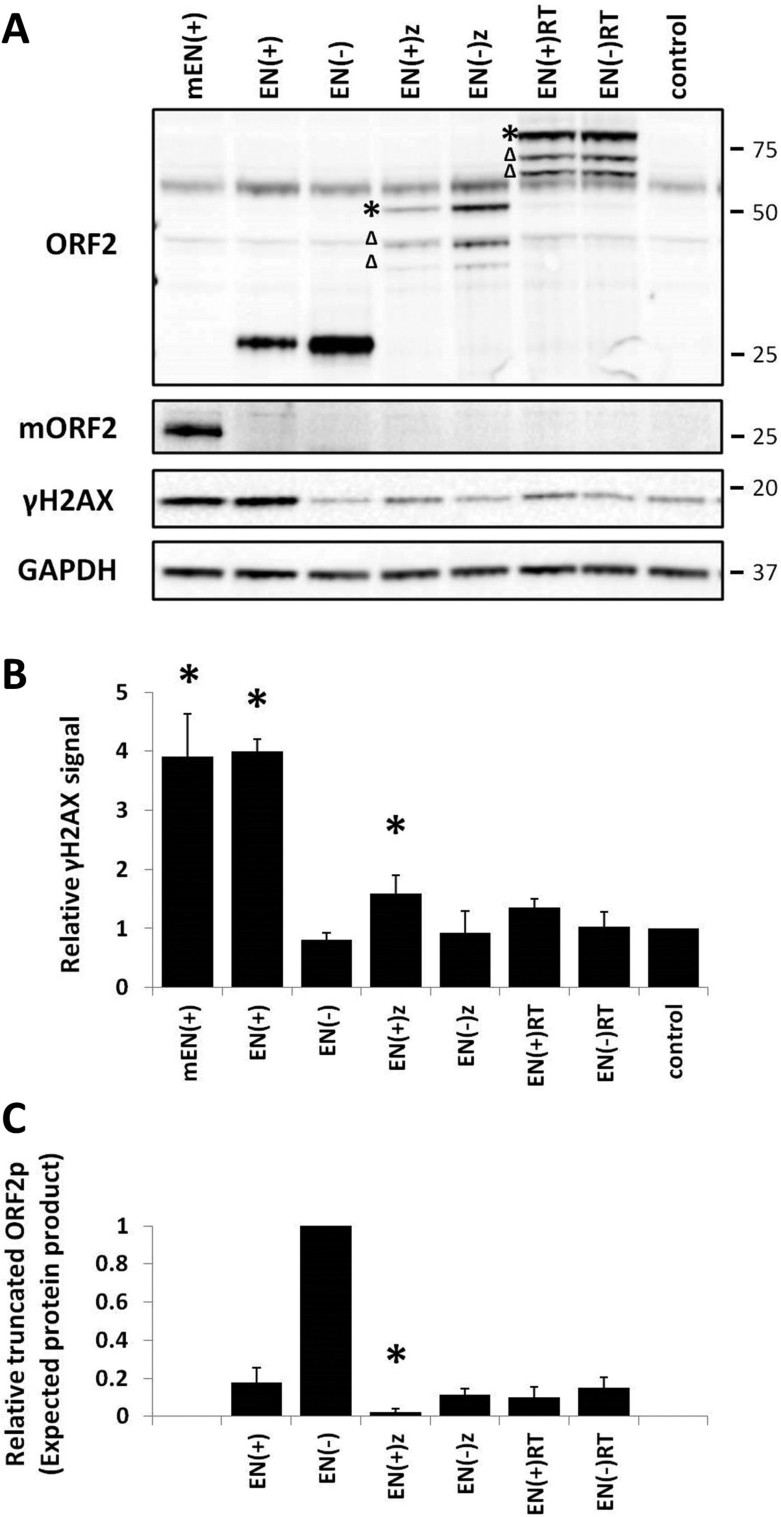
Expression of truncated ORF2 proteins containing a functional endonuclease domain can induce DNA damage. (**A**) Representative western blot of total protein lysates harvested from 293 cells transiently transfected with the indicated truncated ORF2 expression plasmid. Constructs are annotated as having functional endonuclease domains (+) or non-functional mutant endonuclease domains (−). The mEN construct expresses the endonuclease from the mouse L1 ORF2, all others express human L1 ORF2 truncated proteins. Control lanes indicate cells transfected with an empty vector. Molecular size markers are indicated on the right. ORF2 panel, Truncated ORF2 protein was detected with antibodies specific to the human L1 endonuclease domain. Asterisks indicate a band of the expected molecular weight, Δ indicates processed protein products. mORF2 panel, Western blot analysis using an antibody generated against the mouse L1 endonuclease domain. γH2AX, Anti-γH2AX antibodies were used to detect the phosphorylation of histone H2AX as an indication of DNA damage. GAPDH panel, Anti-GAPDH antibodies are used as a loading control. (**B**) Relative γH2AX signals detected in cells after transfection of the truncated ORF2 constructs were normalized to their respective GAPDH loading controls and then expressed as a proportion of the relative signal detected for the empty vector control. Quantitation is from three independent experiments. Asterisks indicate a statistically significant difference between the truncated protein and the empty vector control (*t*-test, *P* ≤ 0.05). (**C**) Relative levels of the expected-sized proteins expressed from the truncated ORF2 constructs were normalized to their respective GAPDH loading controls and then expressed as a proportion of the relative signal detected for EN(−). Quantitation is from three independent experiments. Asterisks indicate a statistically significant difference in the steady-state level of truncated ORF2 proteins containing a functional endonuclease domain in comparison to EN(+) (*t*-test, *P* ≤ 0.05).

To determine whether the variance in the relative toxicity observed after transfection of the different truncated ORF2 constructs is due to the difference in the amount of protein they produce, we analyzed total protein lysates harvested from human 293 cells transiently transfected with the truncated ORF2 expression plasmids. Western blot analysis using antibodies specific to two peptides in the endonuclease domain of the human L1 ORF2 protein (7) detected a single band of the expected molecular weight (∼26 kDa) in the lysates of cells transfected with the EN(+) and EN(−) constructs (Figure 4A, ORF2 panel). As expected, no signal was detected in the samples collected from cells transiently transfected with the mouse mEN(+) expression plasmid or the empty control using these human ORF2-specific antibodies. Western blot analysis identified the expression of multiple L1 endonuclease-related proteins in the lysates from cells transiently transfected with functional or non-functional ENz and ENRT expression plasmids (Figure 4A, ORF2 panel). The highest molecular weight bands detected were consistent with the expected sizes of the ENz (∼54 kDa) and ENRT (∼85 kDa) proteins, as indicated with asterisks in Figure 4A. The presence of the smaller molecular weight bands suggested that the ENz and ENRT proteins may be subjected to proteolytic cleavage. Although it is not clear which specific protein product is responsible for the observed toxicity, the steady-state levels of the expected ∼54 kDa EN(+)z protein, as well as the combined EN(+)z protein products, were both significantly reduced in comparison to EN(+)p (Figure 4C, Supplementary Figure S4). No significant difference was observed in the steady-state levels of EN(+)RT, compared to EN(+), for either the expected ∼85 kDa protein or the combined EN(+)RT protein products (Figure 4C, Supplementary Figure S4).

We also generated polyclonal antibodies specific to the endonuclease domain of the mouse L1 in order to detect expression of mEN(+)p in human cells. Western blot analysis of total protein lysates harvested from transiently transfected 293 cells detected expression of the mouse endonuclease domain (Figure 4A, mORF2 panel). Both the human L1 ORF2 antibody and the mouse ORF2 antibody demonstrated high specificity, as no cross-reactivity was detected with the endonuclease domain from the opposite species (Figure 4A).

### Expression of the L1 ORF2 endonuclease domain induces DNA damage in mammalian cells

Expression of L1 or the L1 ORF2 protein has been reported to induce DSBs in an endonuclease-dependent manner (6). To confirm that the expression of truncated ORF2 proteins containing a functional endonuclease domain causes DNA damage, a γH2AX signal in cells transiently transfected with the truncated ORF2 constructs was used as an indication of DNA damage (54). The strongest γH2AX signal was detected in the total protein lysates from 293 cells transiently transfected with the functional human EN(+) or mouse mEN(+) endonuclease domains (Figure 4A, γH2AX panel). Expression of the functional human EN(+) protein in 293 cells generated over a 5-fold increase in the relative γH2AX signal when compared to the non-functional EN(−) (Figure 4B). Results from western blot analysis of cells transiently transfected with EN(+)z demonstrated a significant, but modest increase in the relative γH2AX signal in comparison to its non-functional counterpart and to the empty plasmid control (Figure 4B). These results are consistent with the observed differences in toxicity caused by transfection of the EN(+) and EN(+)z constructs in 293 cells (Figure 3C, Supplementary Figure S2). Expression of the functional EN(+)RT protein did not generate a significant increase in the relative γH2AX signal in comparison to its non-functional counterpart (Figure 4B), even though transfection of this construct caused significant acute and chronic toxicity in 293 cells (Figure 3C, Supplementary Figure S2).

**Figure 5.**
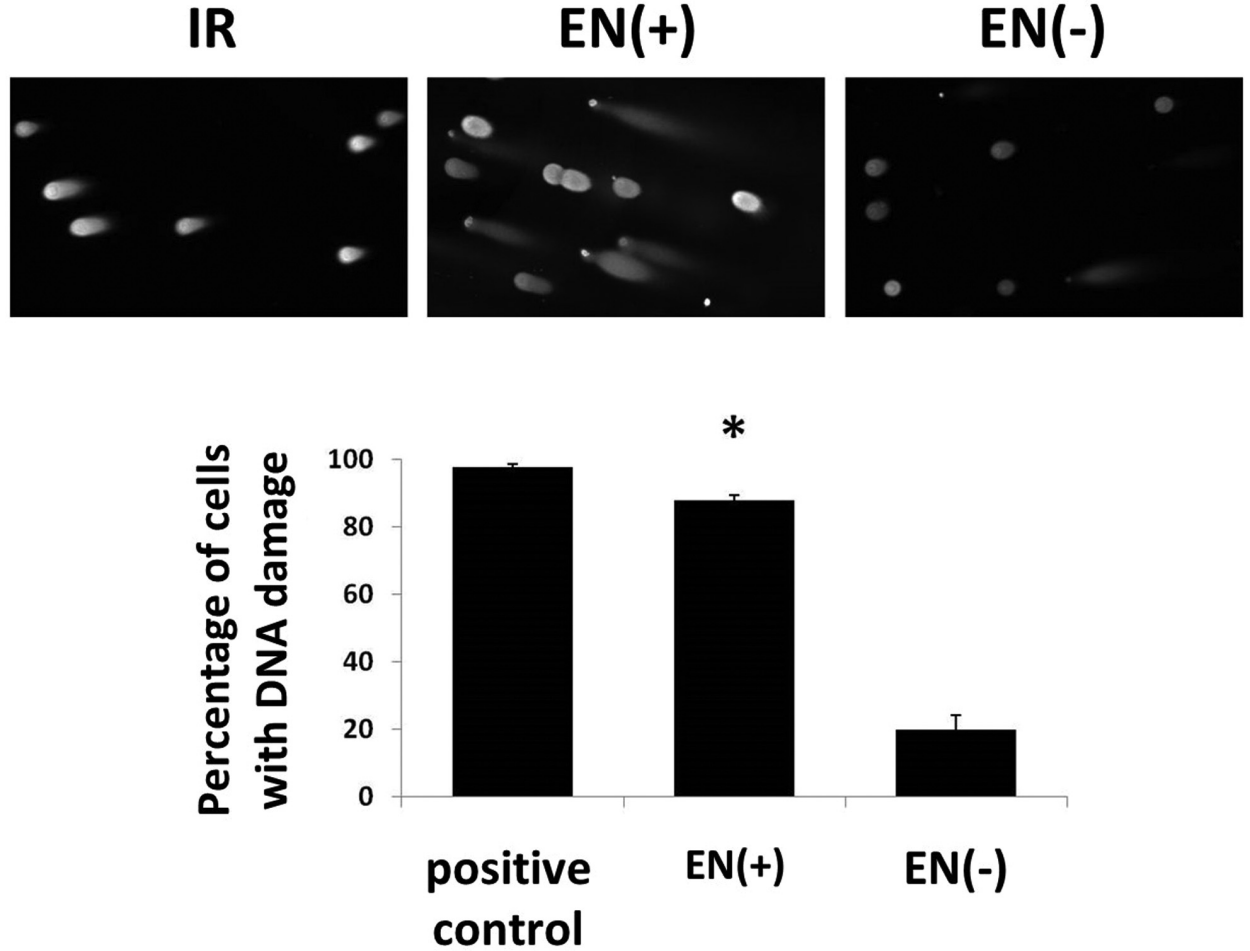
Visualization of DNA damage generated by expression of the L1 endonuclease domain in individual cells. Top, Representative images (100×) from neutral COMET assays in HeLa cells subjected to 5 Gy of ionizing radiation (IR) (left), or transfected with the functional EN(+) domain (middle), or non-functional EN(−) mutant (right). Bottom, Quantitation of the percentage of individual cells exhibiting DNA damage as indicated by the presence of a fragmented DNA ‘tail’ from three independent experiments. Asterisks indicate a statistically significant difference in the percentage of individual cells exhibiting DNA damage after expression of the endonuclease-functional EN(+) in comparison to the non-functional mutant EN(−) (*t*-test, *P* ≤ 0.01).

We further confirmed these data by utilizing the neutral COMET assay to detect DNA damage generated by expression of the EN protein in individual cells. Transient expression of the functional EN(+)p resulted in a 4.4-fold higher proportion of individual cells exhibiting DNA damage, as indicated by the presence of a fragmented DNA ‘tail’, in comparison to its non-functional EN(−) counterpart (Figure 5). The DNA damage, or the cellular response to DNA damage after EN(+) expression, was so devastating that we often observed a wide separation of the comet head and tail, with the two often present in different viewing fields at 100× (Supplementary Figure S5).

**Figure 6.**
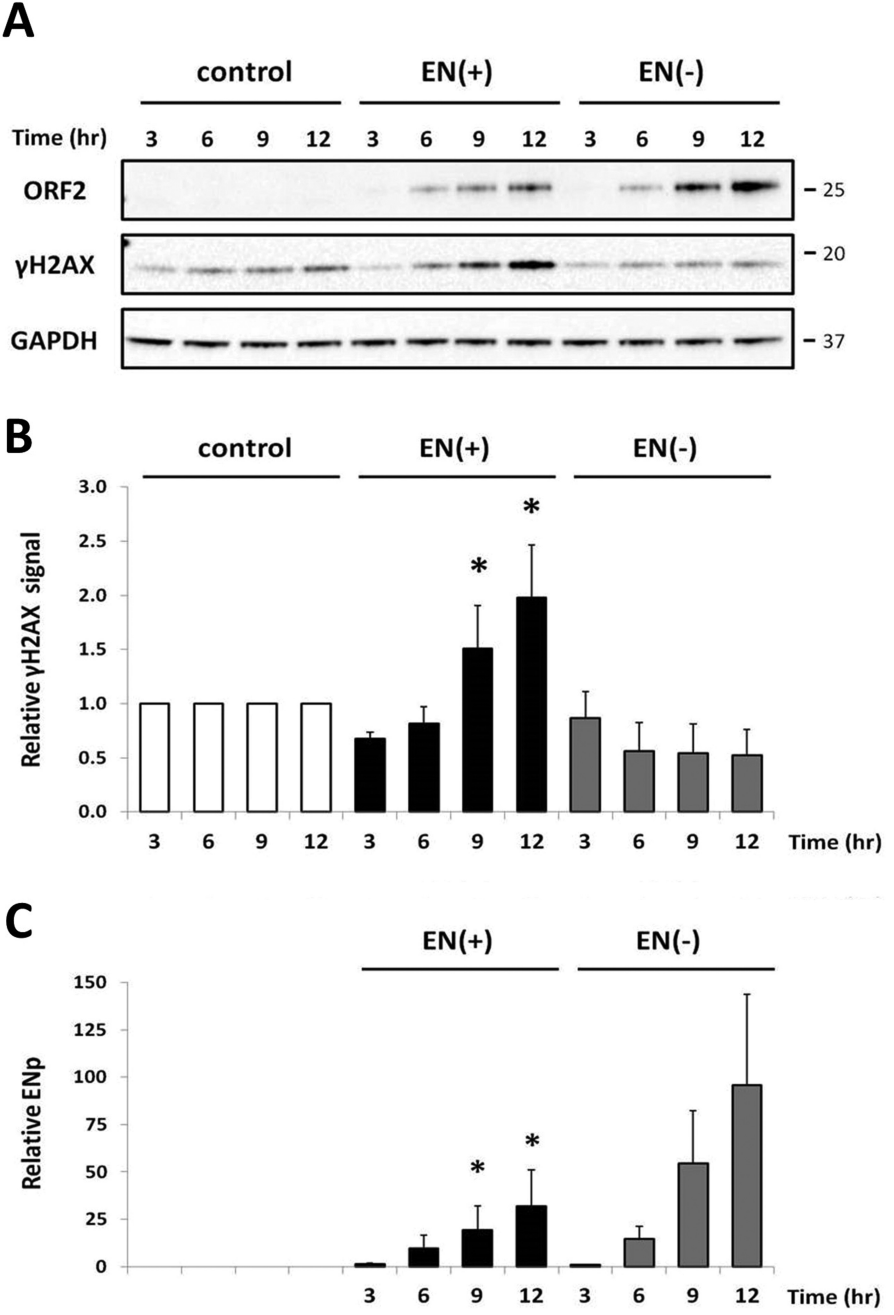
Detection of DNA damage is concurrent with expression of the L1 endonuclease domain. (**A**) Representative western blot analysis of 293 cells transiently transfected with the functional or non-functional endonuclease construct. Control lanes indicate cells transfected with an empty vector. Total protein was harvested at 3, 6, 9 or 12 h post-transfection as indicated. Endonuclease expression was detected with antibodies specific to the human L1 endonuclease domain (ORF2 panel), anti-γH2AX antibodies were used as an indication of DNA damage (γH2AX panel) and anti-GAPDH antibodies were used as a loading control (GAPDH panel). Molecular size markers are indicated on the right. (**B**) Relative levels of γH2AX in cells after expression of EN(+) or EN(−) were normalized to their respective GAPDH loading controls and then expressed as a proportion of the relative signal detected for the empty vector control per time point. Quantitation is from three independent experiments. Asterisks indicate a statistically significant difference between the truncated protein and the empty vector control per time point (*t*-test, *P* ≤ 0.05). (**C**) Relative levels of EN(+) or EN(−) were normalized to their respective GAPDH loading controls and then expressed as a proportion of the relative signal detected for EN(−) at 3 h. Quantitation is from three independent experiments. Asterisks indicate a statistically significant difference between the truncated protein and EN(−) at 3 h (*t*-test, *P* ≤ 0.05).

The DNA damage detected by western blot analysis (Figure 4A and B) and COMET assays (Figure 5) in cells 24 h post-transfection may be a direct result of L1 endonuclease activity, or may reflect the outcome of the cellular response to L1 endonuclease-induced damage (6,29). We conducted a time course experiment in order to investigate the relationship between the timing of endonuclease expression and the detection of DNA damage (Figure 6). Human 293 cells were transiently transfected with constructs expressing the functional or non-functional human L1 endonuclease domain [EN(+) or EN(−)], and total protein lysates were harvested at 3, 6, 9 and 12 h post-transfection. Immunoblot analysis demonstrated that the increase in the γH2AX signal was concurrent with the accumulation of functional EN(+) protein (Figure 6A–C). A significant increase in the relative γH2AX signal was observed beginning at 9 h post-transfection in lysates from cells expressing functional EN(+)p, in comparison to both the non-functional EN(−) and empty control (Figure 6B). As expected, accumulation of the non-functional EN(−)p did not generate a corresponding increase in the γH2AX signal throughout the time course (Figure 6B and C).

Additionally, we conducted a dose curve experiment in which 293 cells were transiently transfected with increasing amounts of the functional and non-functional human EN constructs. Western blot analysis demonstrated that expression of the functional EN(+)p produces a γH2AX signal in a dose-dependent manner, with distinct thresholds for detection of EN and γH2AX (Supplementary Figure S6). We also performed a dose curve experiment in NIH-3T3 cells transiently transfected with the functional and non-functional human EN constructs. Western blot analysis demonstrated that transfection of mouse cells with increasing amounts of the functional human EN(+) expression plasmid generates DNA damage (Supplementary Figure S7), confirming the activity of the human L1 ORF2 endonuclease domain in a non-human mammalian cell line.

### Subcellular localization of truncated ORF2 proteins in mammalian cells

The variation in cellular toxicity and DNA damage generated after expression of the different truncated ORF2 proteins can be partially explained by the differences in their steady-state levels (Figure 4A). However, it is also possible that these proteins differ in their ability to localize to the nucleus, potentially preventing the endonuclease domain from gaining access to its substrate. This consideration is particularly compelling because of the difference in sizes among the truncated ORF2 proteins. The small molecular weight of ENp may allow for passive translocation into the nucleus, while the larger truncated ORF2 proteins such as ENz and ENRT would more likely require active transport into the nucleus (55).

**Figure 7.**
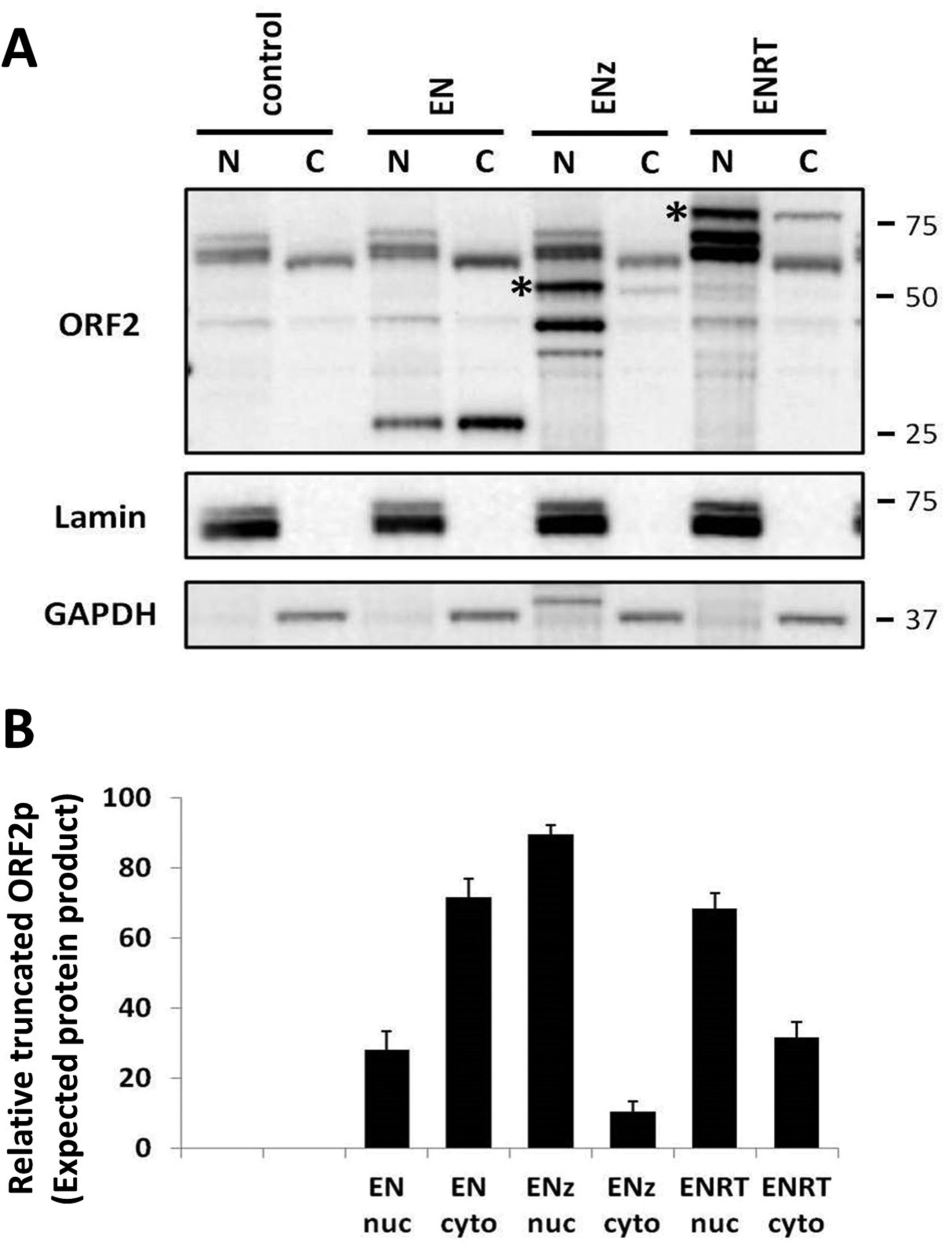
Cellular localization of truncated ORF2 proteins expressed in human cells. (**A**) Representative western blot analysis of nuclear and cytoplasmic fractions from 293 cells transiently transfected with the indicated truncated ORF2 expression plasmid. ORF2 panel, Truncated ORF2 proteins were detected with antibodies specific to the human L1 endonuclease domain; Lamin panel, anti-Lamin A/C antibodies were used as a cellular fractionation control (nuclear); GAPDH panel, anti-GAPDH antibodies were used as a cellular fractionation control (cytoplasmic). Asterisks indicate band of expected molecular weight. Control lanes indicate cells transfected with an empty vector. Molecular size markers are indicated on the right. (**B**) Relative levels of the expected-sized proteins expressed from the truncated ORF2 constructs were normalized to their respective Lamin and GAPDH loading controls, and expressed as a percentage. Quantitation is from three independent experiments.

To investigate the subcellular localization of truncated ORF2 proteins, we examined the nuclear and cytoplasmic fractions from cells transiently transfected with the various truncated ORF2 expression plasmids as previously described (48). Western blot analysis with antibodies specific to the ORF2 endonuclease domain demonstrated that only 28% (±5%) of ENp was localized to the nuclear fraction in 293 cells (Figure 7A and B). The same analysis showed that the expected ∼54 kDa ENz and ∼85 kDa ENRT proteins were predominantly localized to the nucleus, with ∼90% (±3%) and 68% (±4%) respectively, detected in the nuclear fraction (Figure 7A and B, asterisk indicates expected size). This primarily nuclear localization was also observed for all of the faster migrating ENz and ENRT protein products (Figure 7A). In addition, we observed a similar subcellular localization of the truncated ORF2 proteins when expressed in HeLa cells (Supplementary Figure S8). The primarily nuclear localization of the ENz and ENRT proteins suggested that an NLS may exist downstream of the endonuclease domain. Using cNLS Mapper (47), we identified a putative NLS within the z-motif region of ORF2 and generated NLS mutant versions of the ENz and ENRT expression plasmids. The subcellular localization of the ENz and ENRT proteins with mutations in the putative NLS did not change when transiently expressed in 293 cells (Supplementary Figure S9).

### Expression of the ORF2 endonuclease domain from the wild-type L1 sequence is toxic in mammalian cells

**Figure 8.**
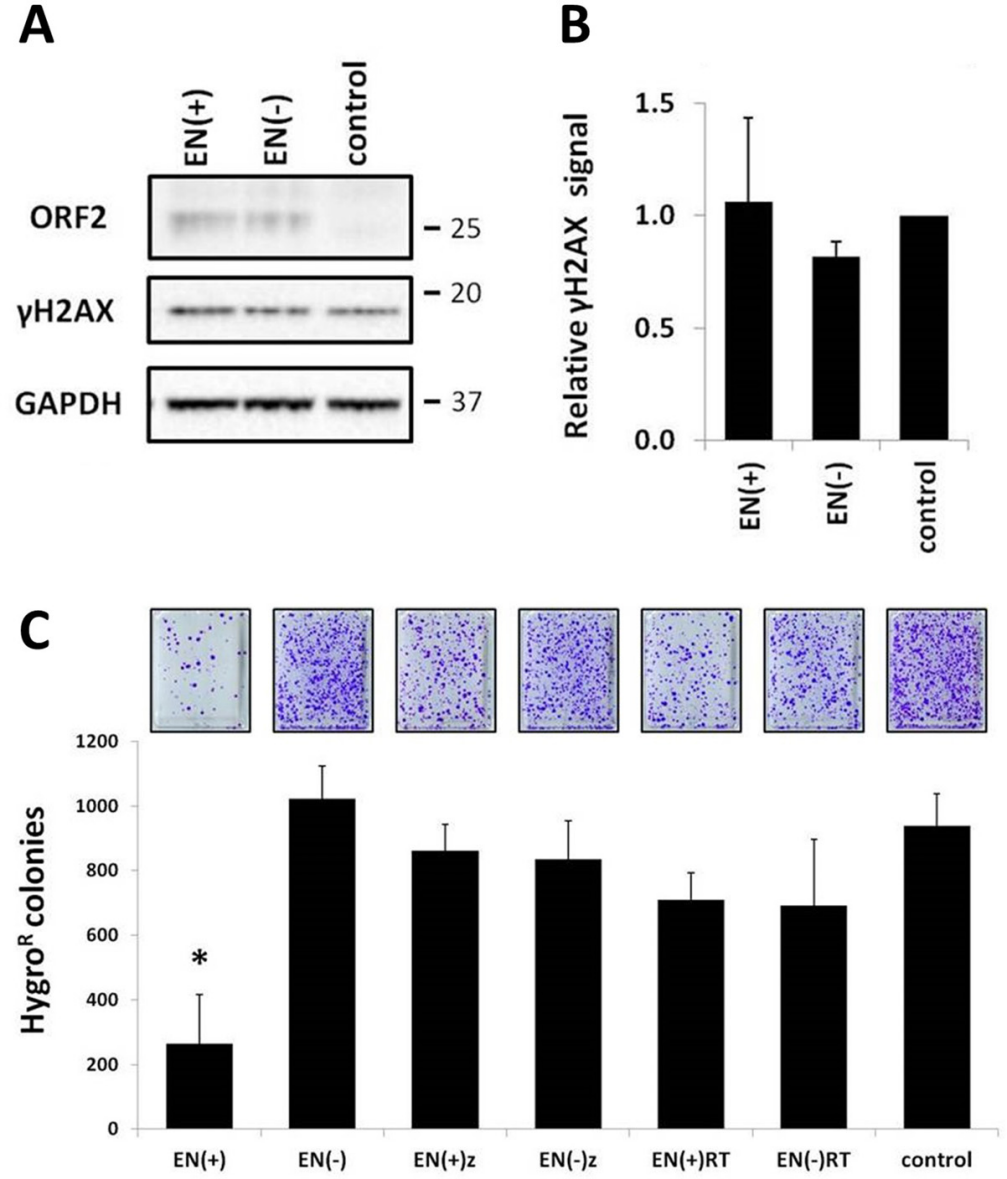
Chronic expression of the ORF2 endonuclease domain from the wild-type L1 sequence is toxic. (**A**) Representative western blot of total protein lysates harvested from 293 cells transiently transfected with the indicated construct. The EN(+) and EN(−) constructs express the functional and non-functional endonuclease domain from the wild-type human L1 ORF2 sequence. Control indicates cells transfected with an empty vector. Endonuclease expression was detected with antibodies specific to the human L1 endonuclease domain (ORF2 panel), anti-γH2AX antibodies were used as an indication of DNA damage (γH2AX panel) and anti-GAPDH antibodies were used as a loading control (GAPDH panel). Molecular size markers are indicated on the right. (**B**) Relative levels of γH2AX in cells after expression of the endonuclease domain were normalized to their respective GAPDH loading controls and then expressed as a proportion of the relative signal detected for the empty vector control. Quantitation is from three independent experiments. (**C**) Results from four independent chronic toxicity assays (29) in 293 cells after stable expression of the indicated truncated ORF2 proteins from wild-type human L1 sequences (*X*-axis). *Y*-axis is the number of Hyg^R^ colonies formed after stable expression of the constructs under Hyg selection for 2 weeks. Constructs are annotated as having functional endonuclease domains (+) or non-functional mutant endonuclease domains (−). Asterisk indicates a statistically significant difference between the truncated protein containing a functional endonuclease and its non-functional counterpart (*t*-test, *P* ≤ 0.01).

Western blot analysis confirmed the expression, and DNA damaging potential, of truncated ORF2 proteins with a functional endonuclease domain (Figures 4A, B and 6). These truncated ORF2 proteins were expressed from plasmids containing codon-optimized L1 sequences, which support much higher levels of protein expression than do wild-type L1 sequences (45). Western blot analysis of lysates harvested from 293 cells transiently transfected with plasmids containing wild-type L1 sequences confirmed low expression of both the functional and non-functional ENp (Figure 8A). We did not detect expression of ENz or ENRT proteins from cells transfected with the truncated wild-type L1 constructs (data not shown). This observation is consistent with the expression patterns observed for the truncated ORF2 proteins produced by codon-optimized plasmids, as ENp had the highest steady-state level (Figure 4A).

Although transient expression of EN(+)p from the wild-type L1 sequence did not generate a robust γH2AX signal (Figure 8A and B), chronic toxicity assays performed in 293 cells demonstrated that the persistent expression of the endonuclease domain from the wild-type L1 sequence is toxic (Figure 8C) (29). Stable transfection of the functional EN(+) construct with wild-type sequence resulted in a ∼75% reduction in the number of Hyg^R^ colonies when compared to its non-functional counterpart (Figure 8C). No significant difference in toxicity was observed after stable transfection of the EN(+)z and EN(+)RT constructs with wild-type sequences in comparison to their respective non-functional counterparts (Figure 8C).

### Cellular toxicity and DNA damage generated by the expression of full-length ORF2 constructs containing premature stop codons

**Figure 9.**
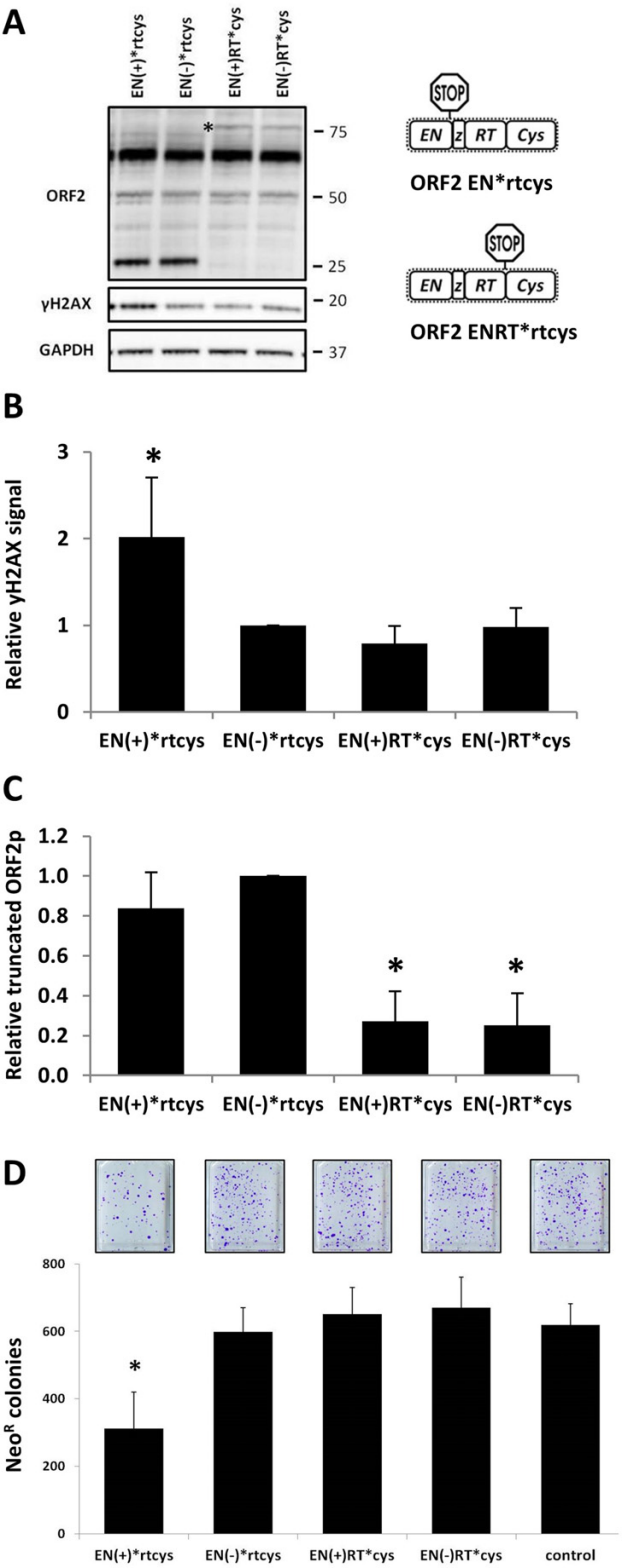
Truncated proteins are expressed from full-length ORF2 sequences containing a premature stop codon. (**A**) Schematic of full-length ORF2 expression constructs containing premature stop codons after the EN or RT domains. Representative western blot analysis of the expression of truncated proteins generated from full-length ORF2 constructs containing premature stop codons in 293 cells. Constructs are annotated as having functional endonuclease domains (+) or non-functional mutant endonuclease domains (−). Blots were probed with the polyclonal antibody generated against the human L1 endonuclease to identify expression of truncated ORF2 proteins (ORF2 panel); anti-γH2AX antibodies were used as an indication of DNA damage (γH2AX panel); and anti-GAPDH as a loading control (GAPDH panel). * indicates band of expected molecular weight. Molecular size markers are indicated on the right. (**B**) Relative levels of γH2AX in cells after expression of the premature stop ORF2 constructs were normalized to their respective GAPDH loading controls and then expressed as a proportion of the relative signal detected for EN(−)*rtcys. Quantitation is from four independent experiments. Asterisks indicate a statistically significant difference between constructs containing a functional endonuclease domain and its non-functional counterpart (*t*-test, *P* ≤ 0.05). (**C**) Relative levels of the truncated proteins expressed from the full-length ORF2 premature stop constructs were normalized to their respective GAPDH loading controls and then expressed as a proportion of the relative signal detected for EN(−)*rtcys. Quantitation is from four independent experiments. Asterisks indicate a statistically significant difference between the EN(+)*rtcys and EN(+)RT*cys constructs (*t*-test, *P* ≤ 0.01). (**D**) Results from four independent acute toxicity assays (6) indicate a significant reduction in the number of G418^R^ colonies (*Y*-axis) formed in 293 cells after transient expression of the indicated ORF2 premature stop constructs (*X*-axis). Asterisks indicate a statistically significant difference between constructs containing a functional endonuclease domain and its non-functional counterpart (*t*-test, *P* ≤ 0.01).

Having found evidence that truncated ORF2 proteins are stable and can retain endonuclease activity in mammalian cells, we expanded upon these findings by using constructs that more closely approximate a biologically relevant scenario. To reproduce retrotransposition-inactivating mutations found within L1 *loci* in the genome, we used site-directed mutagenesis to introduce premature translation termination codons after the endonuclease domain (EN*rtcys) or after the reverse transcriptase domain (ENRT*cys) in a codon-optimized, full-length ORF2 expression plasmid (Figure 9A). Additionally, we generated non-functional versions of the ORF2 premature stop constructs, which contain an inactivating mutation (D205A) in the endonuclease domain (46). Western blot analysis detected expression of truncated ORF2 proteins in lysates collected from cells transiently transfected with constructs containing premature stop codons within the full-length ORF2 sequence (Figure 9A, asterisk indicates truncated protein produced by ENRT*cys). The truncated protein expressed by the EN(+)*rtcys plasmid generated twice as much of a γH2AX signal in 293 cells relative to that of its non-functional counterpart, EN(−)*rtcys (Figure 9A, γH2AX panel, and Figure 9B). There was no significant difference in the γH2AX signal observed in total protein lysates from cells transfected with the EN(+)RT*cys (functional endonuclease) or EN(−)RT*cys (non-functional endonuclease) constructs (Figure 9A and B). The steady-state level of the truncated protein expressed after transfection of the EN(+)RT*cys construct was ∼3-fold less than that produced from the EN(+)*rtcys plasmid (Figure 9C). Consistent with the ability of the endonuclease domain to generate DNA damage, transient transfection of EN(+)*rtcys, but not EN(+)RT*cys, caused a significant amount of toxicity in human 293 cells (Figure 9D). Expression of the EN(+)*rtcys construct caused an ∼45% reduction in G418^R^ colonies when compared to its non-functional endonuclease counterpart or to the empty control.

### Transfection of full-length L1 ORF2 constructs containing premature stop codons supports Alu mobilization

**Figure 10.**
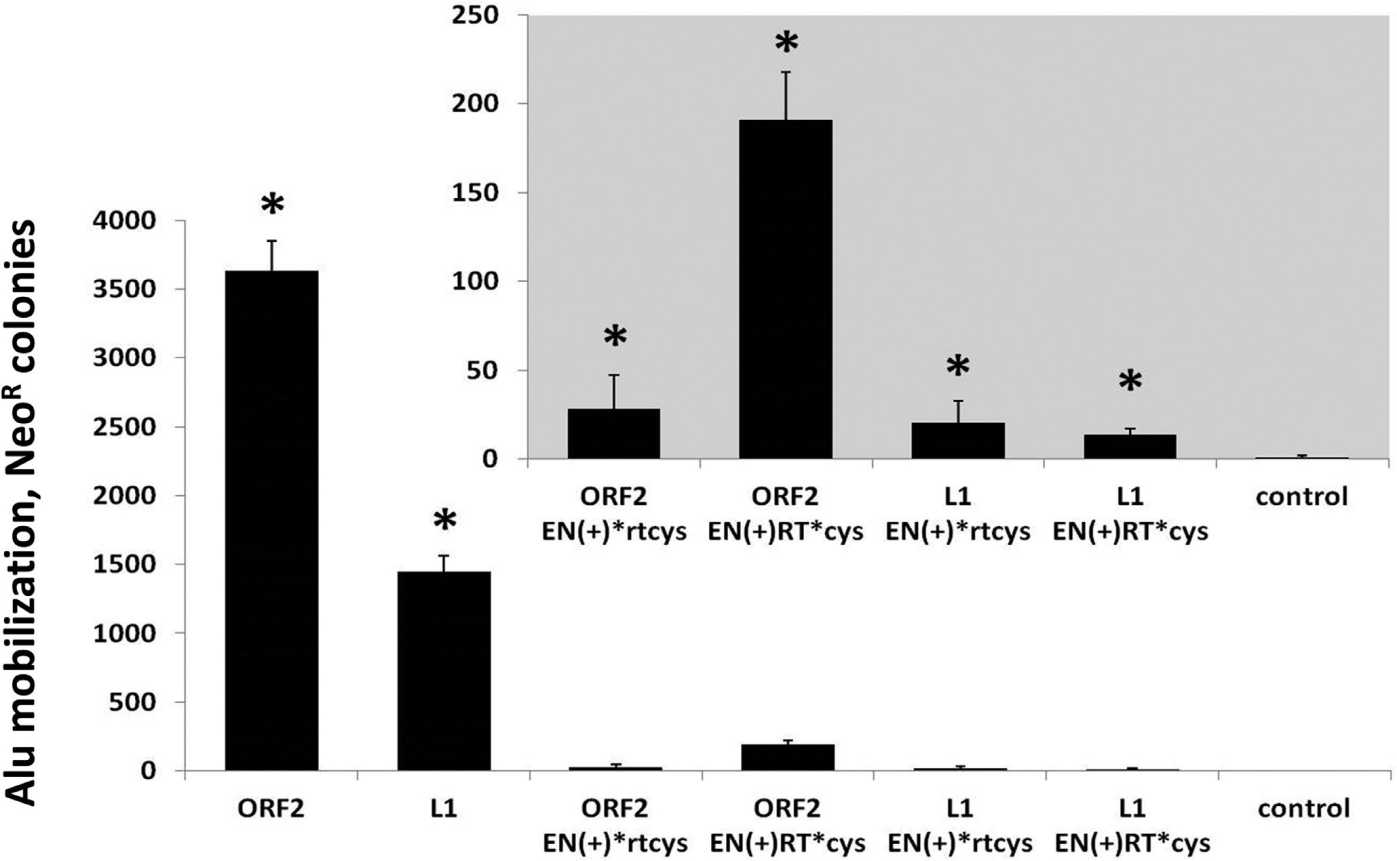
Full-length L1 and ORF2 sequences containing premature stop codons drive Alu mobilization in human cells. Alu retrotransposition assays were performed in HeLa cells, as previously described (15). Full-length L1, ORF2, or L1 and ORF2 constructs containing premature stop codons after the EN domain, or after the RT domain, were used to drive Alu retrotransposition. Control indicates cells transfected with an empty vector and the Alu retrotransposition reporter plasmid. The graph depicts the number of Alu retrotransposition events as represented by G418^R^ colonies (*Y*-axis), and the inset panel shows the same data from the premature stop constructs on an enlarged scale. Asterisks indicate a statistically significant difference in Alu retrotransposition compared to the empty vector control (*t*-test, *P* ≤ 0.05).

In addition to self-retrotransposition and DSB formation, the human L1 retrotransposon is responsible for the mobilization of SINE elements such as Alu (15). Expression of retrotranspositionally-*incompetent* L1 *loci* containing premature stop codons could potentially produce full-length ORF2 proteins, a result of a stop codon readthrough, which may subsequently contribute to Alu retrotransposition. We tested the ability of L1 and ORF2 plasmids containing premature stop codons after the endonuclease domain, or after the reverse transcriptase domain, to drive Alu retrotransposition using a previously established retrotransposition assay (15). Figure 10 demonstrates that transient transfection of the full-length codon-optimized L1 and ORF2 constructs containing premature stop codons supported Alu retrotransposition in HeLa cells, ranging from 10- to 200-fold above the background (gray inset panel). Interestingly, a significant difference in Alu mobilization was observed when retrotransposition was driven by ORF2 EN(+)*rtcys in comparison to ORF2 EN(+)RT*cys. This may be at least partly due to toxicity, as expression of the functional endonuclease domain from the EN(+)*rtcys construct led to significantly more toxicity than that observed in 293 cells after transfection with EN(+)RT*cys (Figure 9D). Overall, these data support the premise that retrotranspositionally-*incompetent* L1 *loci* containing premature stop codons within the ORF2 sequence may contribute to genomic instability through the chronic, low level generation of DSBs and/or Alu mobilization.

## DISCUSSION

L1-driven insertional mutagenesis is known to cause a variety of human genetic diseases; accordingly much of the current L1-related disease research focuses on the identification and quantification of *de novo* L1 retrotransposition events [reviewed in (22,56,57)]. However, this focus on retrotranspositionally-competent L1 *loci* as the only meaningful sources of genomic damage may be limiting. It is estimated that 80–100 of the ∼500,000 fixed L1 *loci* in the human genome are capable of retrotransposition, with only a few highly active L1s responsible for most of the retrotransposition (32,58). Nearly 95% of L1 copies are 5′-truncated and therefore cannot express the full-length mRNA required to produce ORF1 and ORF2 proteins (34,59), both of which are necessary for L1 retrotransposition (31). Most of the remaining ∼6000 full-length L1 elements are retrotranspositionally-*incompetent* due to various mutations such as deletions, frameshifts and premature stop codons (37). However, these types of retrotransposition-inactivating alterations do not necessarily preclude mRNA expression from an L1 *locus*. Indeed, an analysis of expressed sequence tags from a human cell line revealed expression from 410 distinct full-length L1 *loci*, among which only 16 contained the intact ORF1 and ORF2 sequences required for retrotransposition (35). We recovered L1 sequences from a limited RT-PCR analysis in HeLa cells and identified a potential candidate L1 *locus*, which may produce a truncated ORF2 protein (Supplementary Table S1). Future exhaustive analysis of the L1 transcriptome, utilizing advanced methods capable of unambiguously identifying authentic L1 transcripts, will determine the repertoire of defective L1 *loci*, which may have the potential to contribute to genomic instability.

The exact mechanism of L1 ORF2 translation from a bicistronic mRNA is not fully understood, and furthermore may differ between the mouse and human L1 elements (51,60). It has been reported that L1 *loci* with inactivating point mutations or deletions within ORF1 maintain ORF2 translation, provided the ORF1p sequence remains in-frame (15). In contrast, the introduction of a premature stop codon in the ORF1 sequence significantly reduces ORF2p expression from the full-length human L1 element (50,51). Thus, efficient expression of truncated ORF2 proteins would require an intact ORF1 and an ORF2 sequence with at least one premature stop codon. We identified 92 full-length retrotranspositionally-*incompetent* L1 *loci* in the human genome fitting these criteria, and roughly 500 in the mouse genome (Figure 2). Based on the location of the first premature stop codon within the ORF2 sequence, truncated ORF2 proteins of various lengths could be generated from these retrotranspositionally-*incompetent* L1 *loci*, the majority of which would contain an intact endonuclease domain (Figure 2). Although the L1 endonuclease domain expressed alone is functional when purified from bacterial and insect cells (24,36), little is known about its stability and activity in mammalian cells, particularly outside the context of the full-length ORF2p. Since genomic damage and toxicity resulting from the expression of L1 is largely dependent upon the endonuclease activity of the full-length ORF2p (6,9,29), we investigated whether truncated ORF2 proteins containing an intact endonuclease domain were functional and capable of causing DNA damage in mammalian cells.

Our results from toxicity assays and western blot analyses demonstrate that truncated ORF2 proteins containing a functional endonuclease domain vary in their expression, nuclear localization and ability to cause DNA damage and toxicity in mammalian cells (Figures 3–7). Among the three truncated ORF2 proteins tested in this study (EN, ENz and ENRT), expression of ENp generated the highest γH2AX signal and consequently caused the most toxicity (Figures 3 and 4). However, all of the truncated ORF2 proteins containing a functional endonuclease domain were toxic to varying degrees in comparison to their endonuclease-mutant counterpart (Figure 3C). The ability of the L1 endonuclease to induce DNA damage and toxicity is not unique to the human enzyme, as expression of the mEN(+)p in human cells generated a γH2AX signal comparable to that of the one induced by expression of the human EN(+)p (Figure 4A and B). Additionally, both chronic and acute expression of the mouse ENp caused a significant amount of toxicity, similar to the results observed with the human ENp (Figure 3C, Supplemental Figures S2–S3). These results are consistent with the evolutionary conservation of the human and mouse L1 endonuclease sequence and its function in the L1 replication cycle (61). They also demonstrate that the ability of mouse or human ENp to induce DNA damage is not constrained to the cellular environment of its species origin. These results provide experimental verification that expression of the L1 endonuclease domain alone (i.e. outside of the context of the full-length ORF2 protein) results in DNA damage and toxicity in a mammalian cellular environment. Though it would be premature to assume that any truncated ORF2 protein containing a functional endonuclease domain will be stable and toxic, these data demonstrate that toxicity was caused by the expression of ORF2 proteins truncated at these specific domain boundaries (EN, ENz, ENRT) (Figure 3).

One noticeable difference in the expression patterns detected among the three truncated ORF2 proteins was the presence of multiple faster-migrating bands in the lysates of human cells transfected with the ENz or ENRT plasmids (Figure 4A). Because of the position of the epitopes recognized by the L1 endonuclease-specific antibody, these data suggest that cleavage of the ENz and ENRT proteins may occur either within the first 154 amino acids or at the C-terminus. Several highly conserved residues that are critical for endonuclease function are located in the N-terminus (particularly N14 and E43) (24,62). Therefore, a loss of endonuclease activity is expected with the removal of conserved amino acids from the N-terminus (24,62), which suggests that some or all of the smaller ENz and ENRT bands may be non-functional (Figure 4A and B). However, if any of these faster migrating bands represent ENz or ENRT products lacking C-terminal sequences, they could be responsible for the γH2AX signal and toxicity observed in transfected cells. As expected, expression of the truncated ORF2 proteins containing a non-functional endonuclease domain mimicked the expression patterns of their respective functional counterparts, but did not result in DNA damage or toxicity (Figures 3 and 4).

Despite its relatively small size (∼26 kDa), the ENp localized predominantly to the cytoplasm, which is remarkable considering the DNA damage and toxicity that it caused (Figures 3,4 and 7). In contrast, the larger ENz (∼54 kDa) and ENRT (∼85 kDa) proteins localized predominantly to the nucleus. Previous studies have identified a nucleolar localization signal located between amino acids 53 and 71 of the human ORF2 protein (63). Although the protocol used here for subcellular fractionation cannot distinguish between nuclear and nucleolar compartments, we observed predominantly nuclear localization for the larger ENz (∼54 kD) and ENRT (∼85 kD) proteins, but not for ENp (∼26 kDa), suggesting the presence of a possible additional NLS downstream of the endonuclease domain in these truncated proteins (Figure 7). Mutation of a predicted NLS located in the z-motif region of ORF2 did not change the subcellular localization of ENz or ENRT proteins (Supplementary Figure S9). This result suggests the possibility that other *cis*-acting signals within these truncated proteins could be targeting them to the nucleus.

Our findings that truncated ORF2 proteins are stable and active in mammalian cells provided justification for testing constructs that would more closely mimic retrotranspositionally-*incompetent* L1 *loci* in the genome. We expressed both truncated EN and ENRT proteins in cultured cells transfected with plasmids containing full-length codon-optimized ORF2 sequences with premature stop codons at the end of the endonuclease or reverse transcriptase domains (Figure 9A). Although only the functional ENp expressed from EN(+)*rtcys generated detectable DNA damage and caused cellular toxicity (Figure 9B and D), the endonuclease-functional EN(+)RTp expressed from the EN(+)RT*cys construct may also cause biologically relevant damage, but at a level below the sensitivity of our assays. This premise is supported by the results from the time course and dose curve experiments, as an above background γH2AX signal was not observed until a threshold amount of endonuclease had accumulated (Figure 6, Supplementary Figure S6). Similarly, expression of the functional EN(+)p from the EN(+) construct containing wild-type L1 sequence did not generate a significant γH2AX signal, likely due to the overall low level of protein production (Figure 8B). However, chronic expression of the wild-type EN(+)p resulted in a significant amount of toxicity in 293 cells (Figure 8C). These data have important biological implications, as they indicate that expression of some truncated ORF2 proteins retaining a functional endonuclease could be a meaningful source of genomic instability, even if their expression is not readily detectable. This notion may be particularly relevant to human malignancies and aging, as L1 expression is typically upregulated in transformed cells (10–14), and chronic, low level L1 expression is occurring over the life-span of the organism (9). It is important to acknowledge that our truncated and premature stop ORF2 constructs, many of which were codon-optimized, were all driven by a CMV promoter and therefore express much higher amounts of protein in comparison to endogenous levels produced from a full-length bicistronic L1 *locus*. As with many, if not all experimental systems, it is challenging to extrapolate from the results of a short-term experiment to the effects of lifelong exposure to low levels of L1 expression; however, these findings provide a proof of principle that retrotranspositionally-*incompetent* L1 *loci* may contribute to L1-induced genomic instability.

Additionally, transient transfection of full-length L1 and ORF2 expression constructs containing premature stop codons after the endonuclease or reverse transcriptase domains drove Alu retrotransposition, though at low levels (Figure 10). The observed Alu mobilization is likely due to low level expression of full-length ORF2p resulting from readthrough of the premature stop codon. Previous reports have demonstrated that an L1 ORF2 protein containing functional reverse transcriptase and Cys domains is required for Alu mobilization, and expression of the full-length ORF2p by readthrough of a premature stop codon would fulfill this necessary condition (15). These data suggest that expression of retrotranspositionally-*incompetent* L1 *loci* containing premature stop codons in the ORF2 sequence may promote low levels of genomic instability through Alu retrotransposition. Notably, both ORF2 and L1 expression plasmids containing a premature stop codon at the end of the endonuclease domain supported less Alu mobilization than those containing a stop codon at the end of the reverse transcriptase domain (Figure 10). We expect that this difference is most likely due to the elevated toxicity associated with expression of the functional ENp in comparison to ENRTp (Figure 9). While the premature stop constructs were inefficient in driving Alu retrotransposition in comparison to the full-length L1 and ORF2 (Figure 10), these data represent an additional plausible explanation for the highly successful occupation of the human genome by Alu elements in conjunction with previously reported models such as the SRP (reviewed in 64), PABP (reviewed in 65) and L1 splicing models (9,66), as well as the waves of Alu retrotransposition throughout primate evolution (65,67–70).

Overall, this study provides experimental evidence supporting the concept that a subset of retrotranspositionally-*incompetent* L1 *loci*, previously considered to be innocuous, may be capable of causing cellular toxicity and genomic instability *via* the generation of DNA DSBs and Alu mobilization (Figure 1). The extent of DNA damage and retrotransposition caused by expression of such *loci* may vary depending upon the position of the premature stop codon within the ORF2 sequence. Taken together, these findings increase the number of potential L1 *loci* relevant to human health and highlight an overlooked source of chronic genomic instability associated with retrotranspositionally-*incompetent* L1 *loci*.

## SUPPLEMENTARY DATA

Supplementary Data are available at NAR Online.

SUPPLEMENTARY DATA
