# Supplementary material for: Potential for genomic instability associated with retrotranspositionally-incompetent L1 loci
Source: Nucleic Acids Res. 2014 Aug 20;42(16):10488–502. doi: 10.1093/nar/gku687 (PMC4176336; doi:10.1093/nar/gku687)
Supplement: SUPPLEMENTARY DATA [file supp_42_16_10488__index.html]

Potential for genomic instability associated with retrotranspositionally-incompetent L1 loci — Potential for genomic instability associated with retrotranspositionally-incompetent L1 loci — SUPPLEMENTARY DATA 

# Potential for genomic instability associated with retrotranspositionally-*incompetent* L1 *loci*

## SUPPLEMENTARY DATA

**Files in this Data Supplement:**

- SUPPLEMENTARY DATA
