# Supplementary material for: Potential for genomic instability associated with retrotranspositionally-incompetent L1 loci
Source: Nucleic Acids Res. 2014 Aug 20;42(16):10488–502. doi: 10.1093/nar/gku687 (PMC4176336; doi:10.1093/nar/gku687)
Supplement: SUPPLEMENTARY DATA [file supp_gku687_nar-01354-a-2014-File012.pdf]

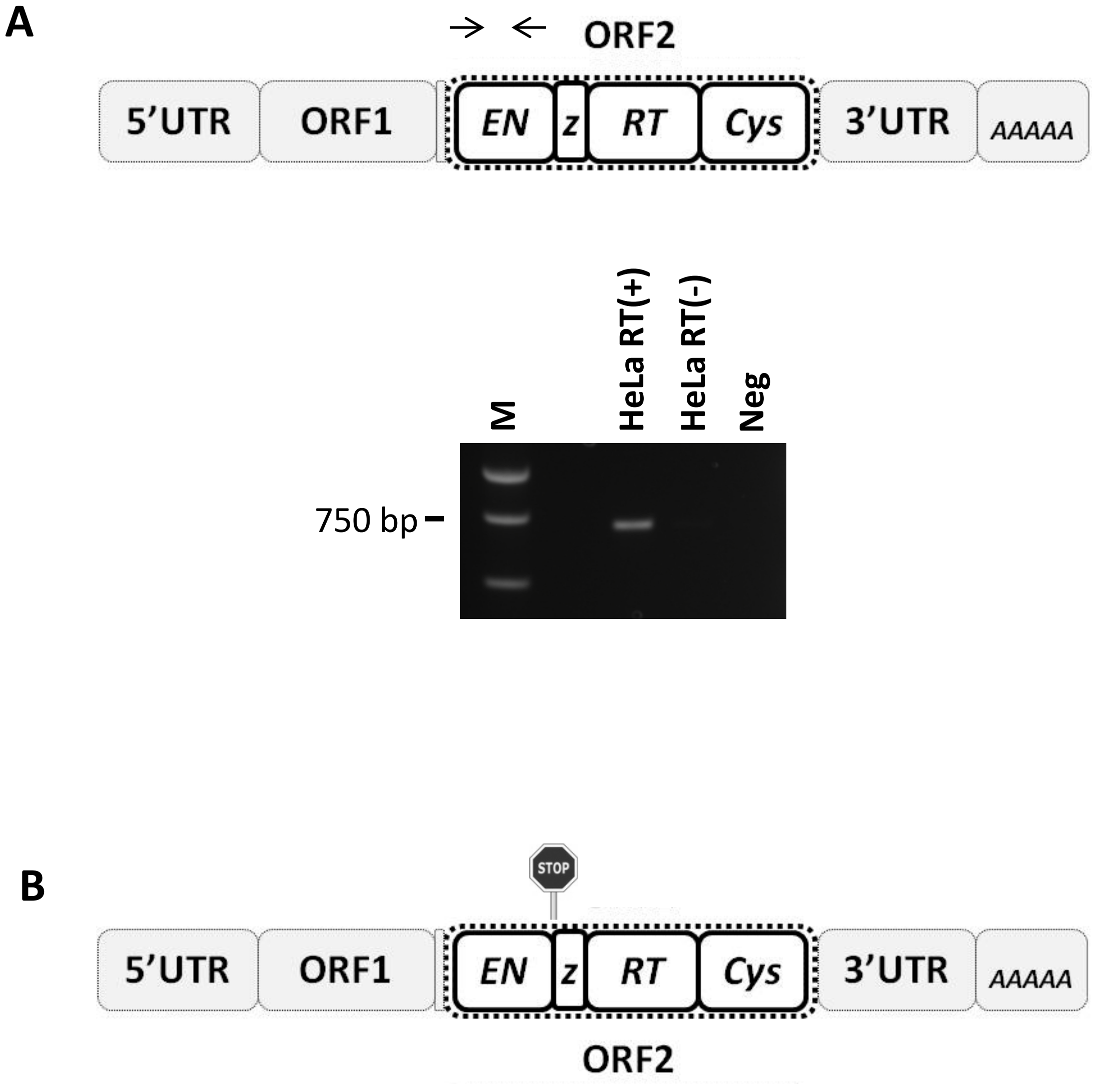

Supplementary Figure S1. Expression of endogenous L1 ORF2 products in HeLa cells. **(A)** RT-PCR analysis of endogenous L1 expression in HeLa cells using primers as indicated by arrows on the L1 schematic. RT(+) and RT(-) indicate reactions with and without reverse transcriptase, respectively. Negative indicates the control PCR reaction without template. **(B)** Schematic of a recovered transcript mapping to a full-length retrotranspositionally-*incompetent* L1 element with the potential to produce a truncated ORF2 protein. This L1 *locus* contains an intact ORF1 with a stop codon located at ORF2 amino acid 377, see Supplementary Table S1 for genomic location. Approximate location of the premature stop codon within ORF2 is indicated by the icon.

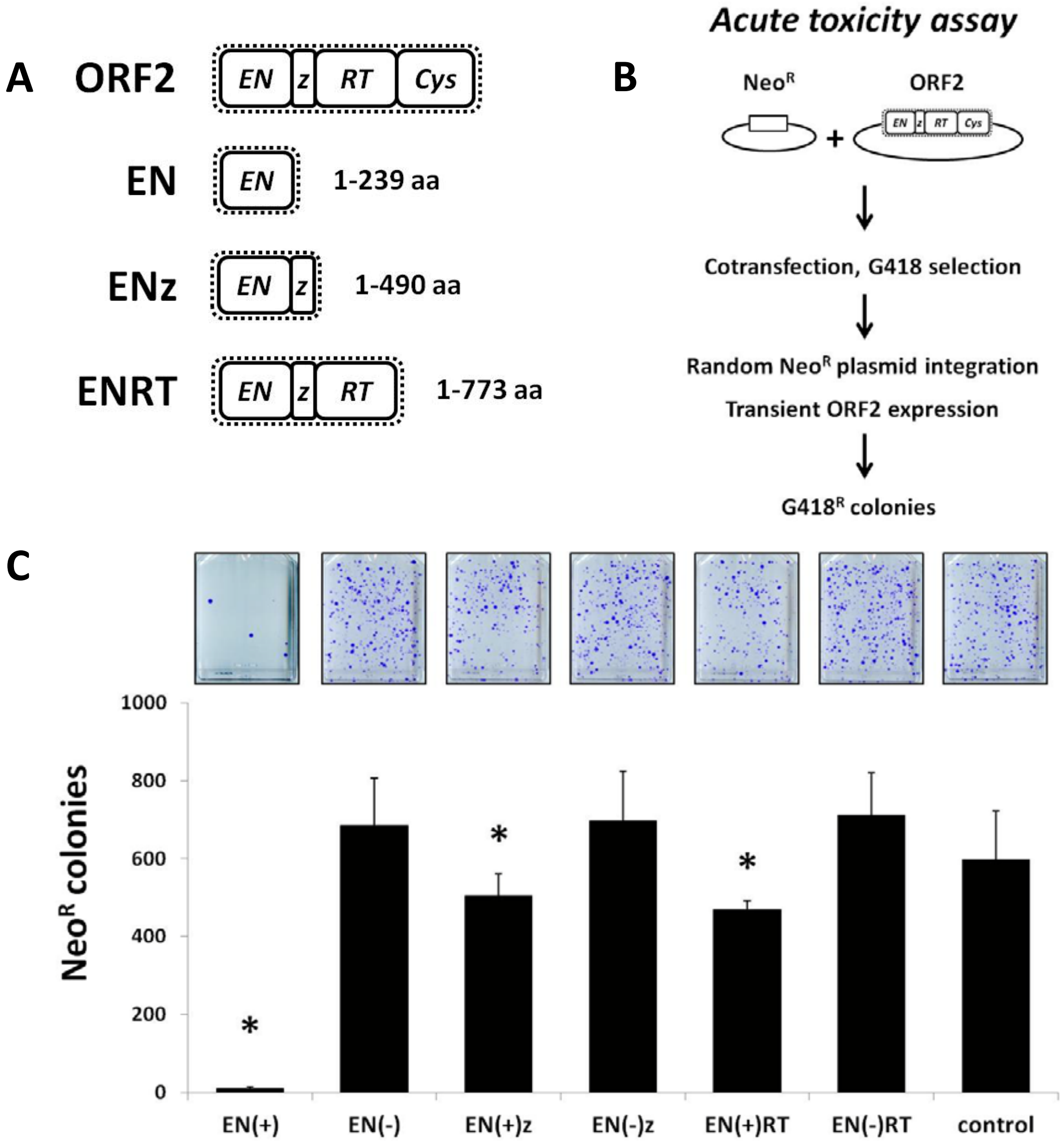

Supplementary Figure S2. Acute toxicity resulting from the transient expression of truncated ORF2 constructs in human cells. (A) Schematic representation of the truncated ORF2 constructs. **(B)** Experimental approach for the acute toxicity assay: Cells are cotransfected with a Neo^R expression vector and the truncated ORF2 construct. Colony formation was assayed after 2 weeks under G418 selection and used as a measure of toxicity. **(C)** Results from four independent acute toxicity assays indicate a significant reduction in the number of $G418^R$ colonies (Y-axis) formed in 293 cells after transient expression of the indicated truncated human ORF2 construct (X-axis). Control indicates cells transfected with an empty vector. Asterisks indicate a statistically significant difference between the functional and non-functional counterpart (t-test, $p \leq 0.05$).

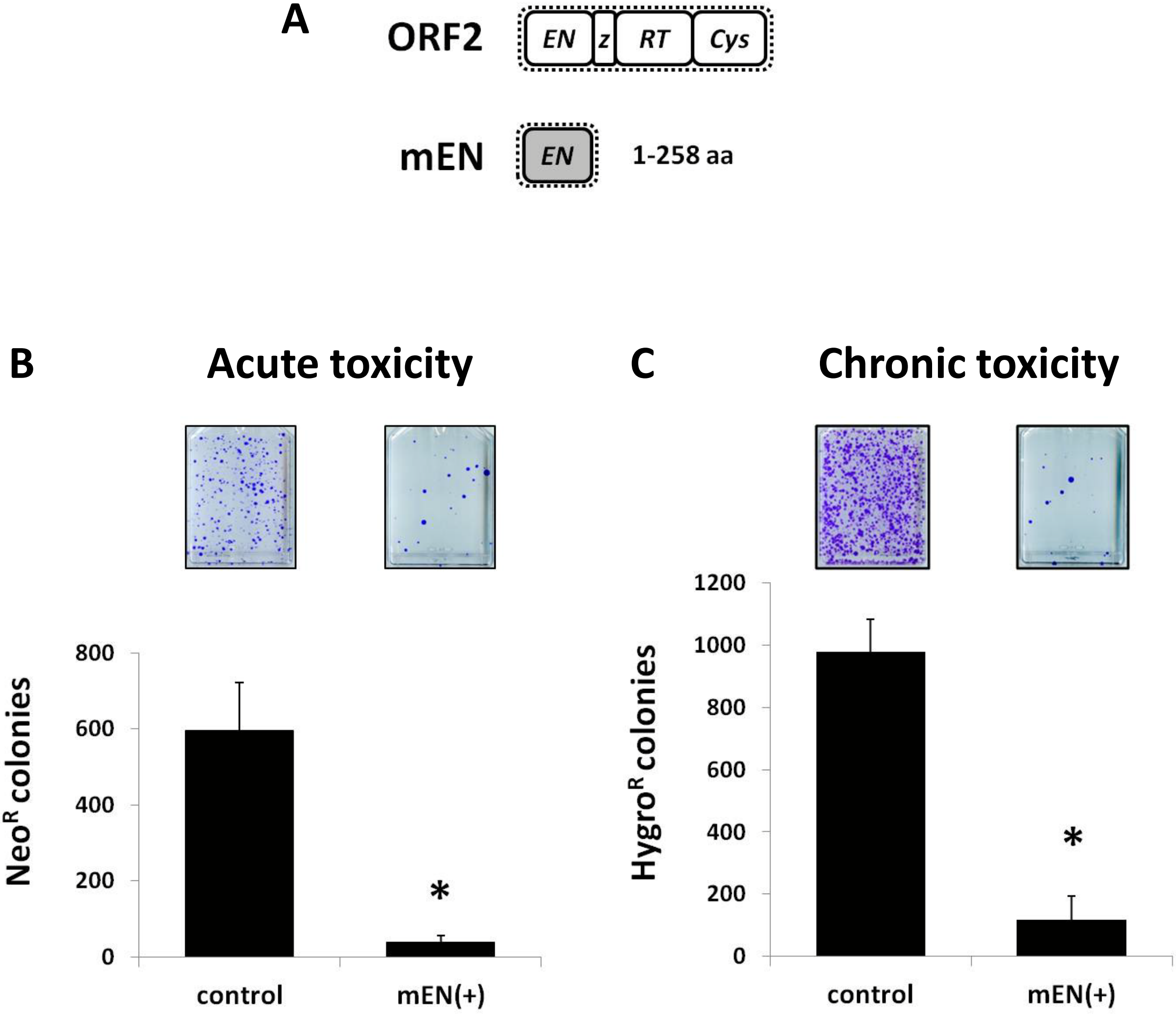

Supplementary Figure S3. Acute and chronic expression of the mouse L1 endonuclease domain causes toxicity in human cells. (A) Schematic representation of the truncated mouse L1 endonuclease construct. **(B)** Results from four independent acute toxicity assays after transient expression of the mouse mEN(+) protein in 293 cells. **(C)** Results from four independent chronic toxicity assays after stable expression of the mouse mEN(+) protein in 293 cells. Asterisks indicate a statistically significant difference between the truncated protein and the empty vector control (t-test, $p \leq 0.01$).

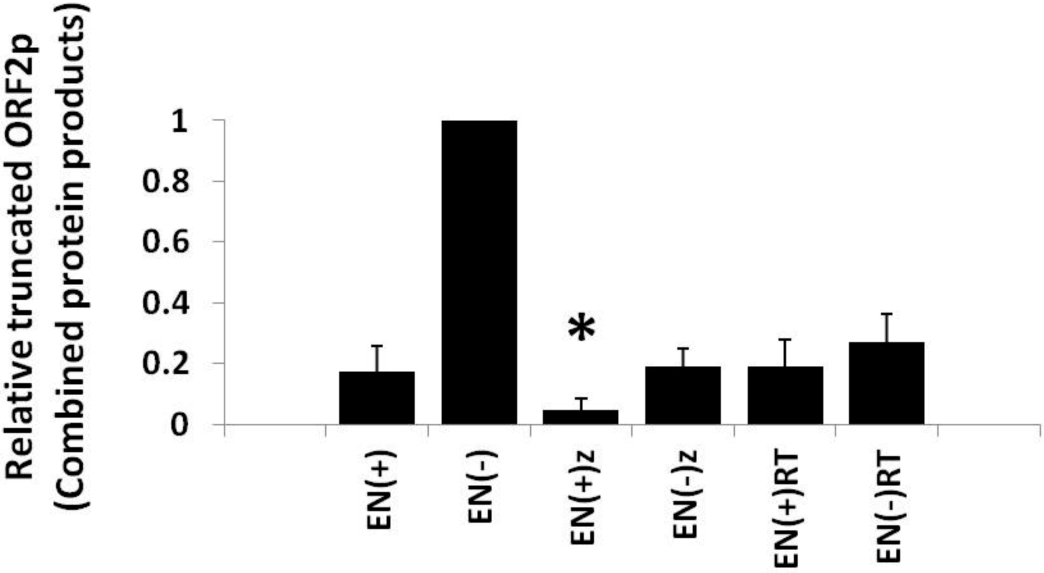

Supplementary Figure S4. Steady-state expression levels of truncated ORF2 proteins in transfected mammalian cells. Further quantitation of protein expression from the western blot experiments in Figure 4. Relative levels of the combined truncated ORF2 proteins detected in 293 lysates harvested after transient transfection of the truncated ORF2 constructs were normalized to their respective GAPDH loading controls and then expressed as a proportion of the relative signal detected for EN(-). Quantitation is from three independent experiments. Asterisk indicates a statistically significant difference in the steady-state level of protein detected in comparison to EN(+) (t-test, $p \leq 0.05$).

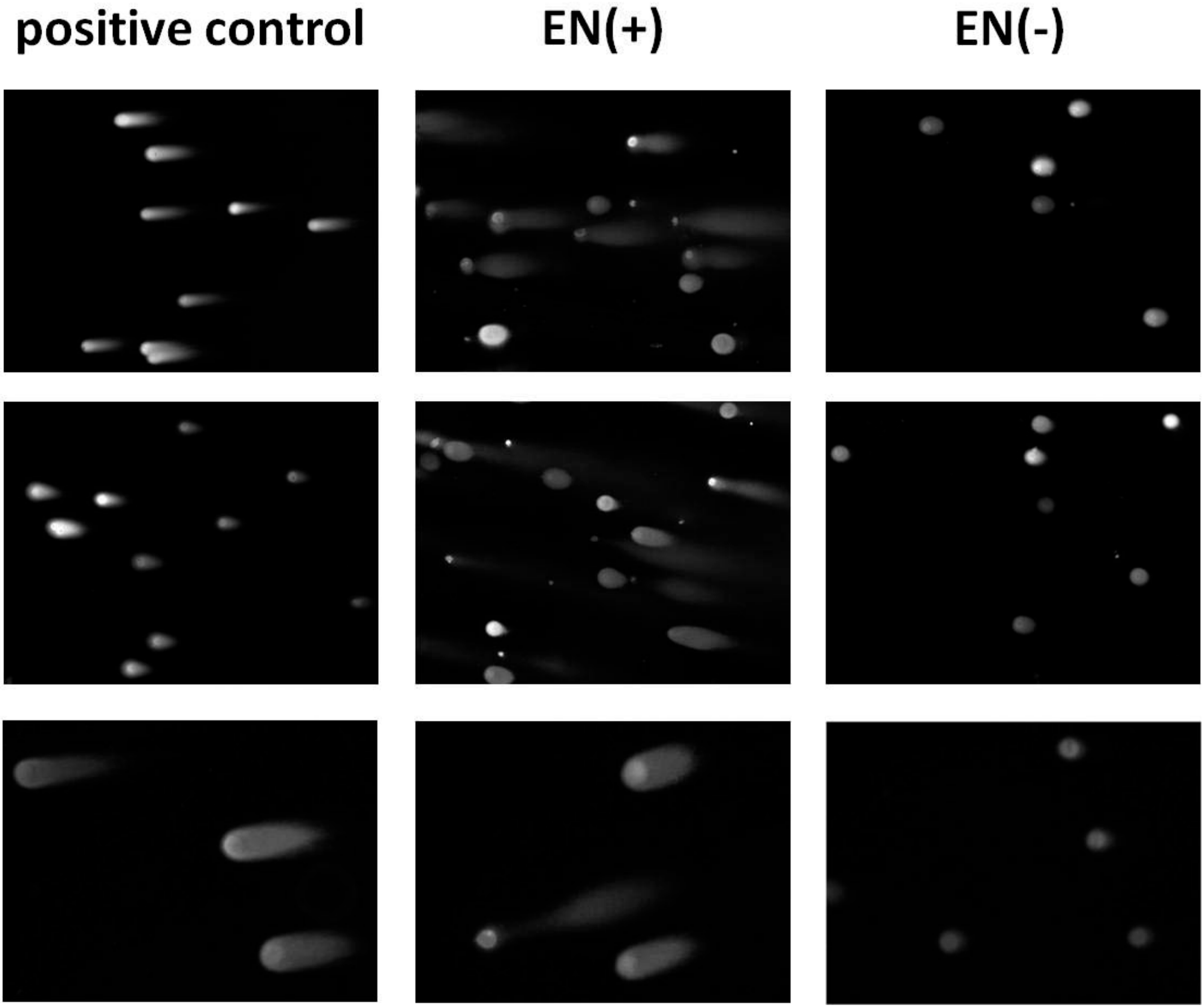

Supplementary Figure S5. COMET assays reveal DNA damage generated by expression of the L1 endonuclease domain. Additional representative images from neutral COMET assays in HeLa cells subjected to ionizing radiation (left column), or transfected with the functional EN(+) (middle), or non-functional EN(-) mutant (right). Images in the top two rows are magnified x100 and images in the bottom row are magnified x200.

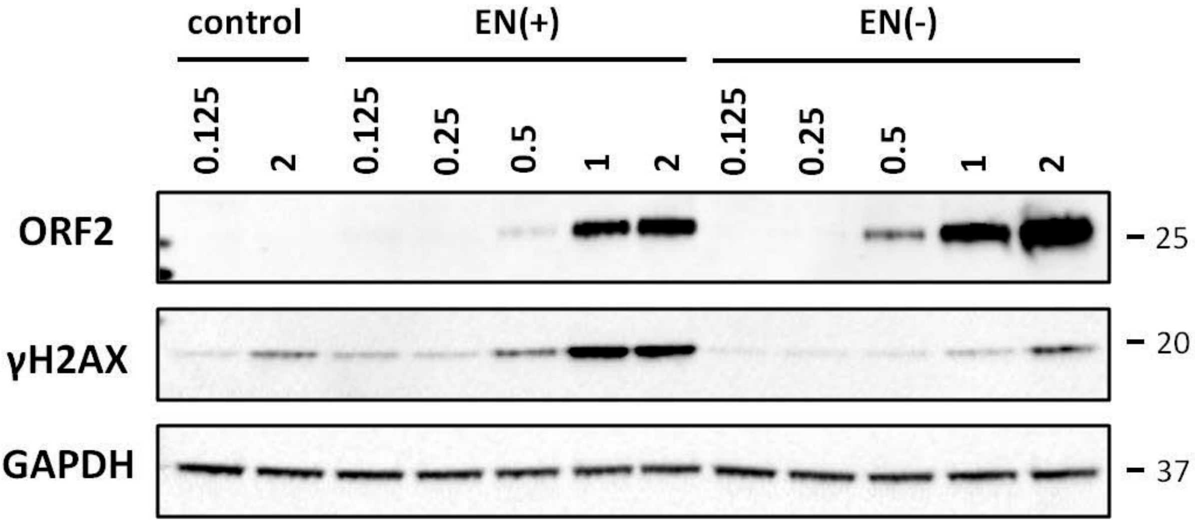

Supplementary Figure S6. Expression of the human L1 endonuclease domain causes DNA damage in a dose-dependent manner. Representative western blot of total protein lysates harvested from 293 cells transiently transfected with the functional or non-functional endonuclease domain. The amount of plasmid DNA transfected (µg) is indicated across the top. Control lanes indicate cells transfected with an empty vector. Endonuclease expression was detected with antibodies specific to the human L1 endonuclease domain (ORF2 panel), anti-γH2AX antibodies were used as an indication of DNA damage (γH2AX panel), and anti-GAPDH antibodies were used as a loading control (GAPDH panel). Molecular size markers are indicated on the right.

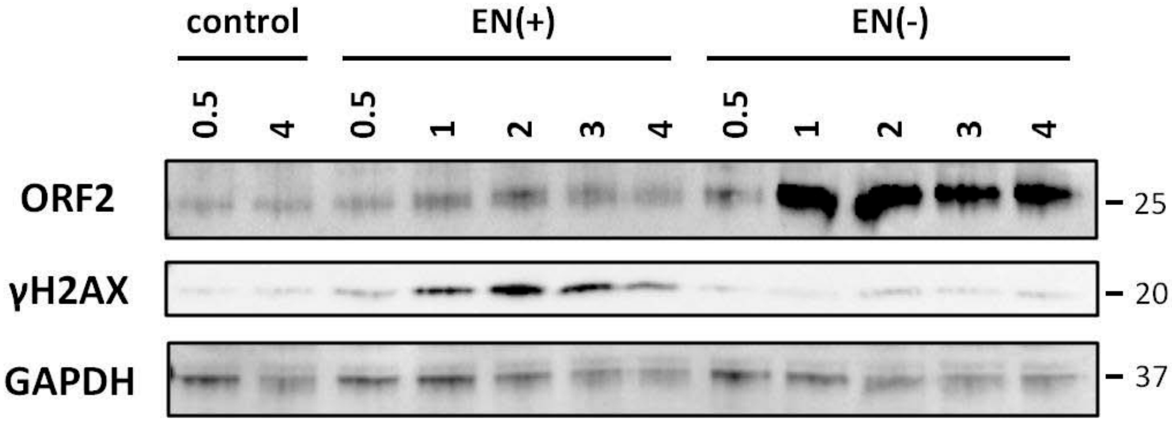

Supplementary Figure S7. Expression of the human L1 endonuclease domain in mouse cells causes DNA damage. Representative western blot of total protein lysates harvested from NIH-3T3 cells transiently transfected with the functional or non-functional human endonuclease domain. The amount of plasmid DNA transfected (µg) is indicated across the top. Control lanes indicate cells transfected with an empty vector. Endonuclease expression was detected with antibodies specific to the human L1 endonuclease domain (ORF2 panel), anti-γH2AX antibodies were used as an indication of DNA damage (γH2AX panel), and anti-GAPDH antibodies were used as a loading control (GAPDH panel). Molecular size markers are indicated on the right.

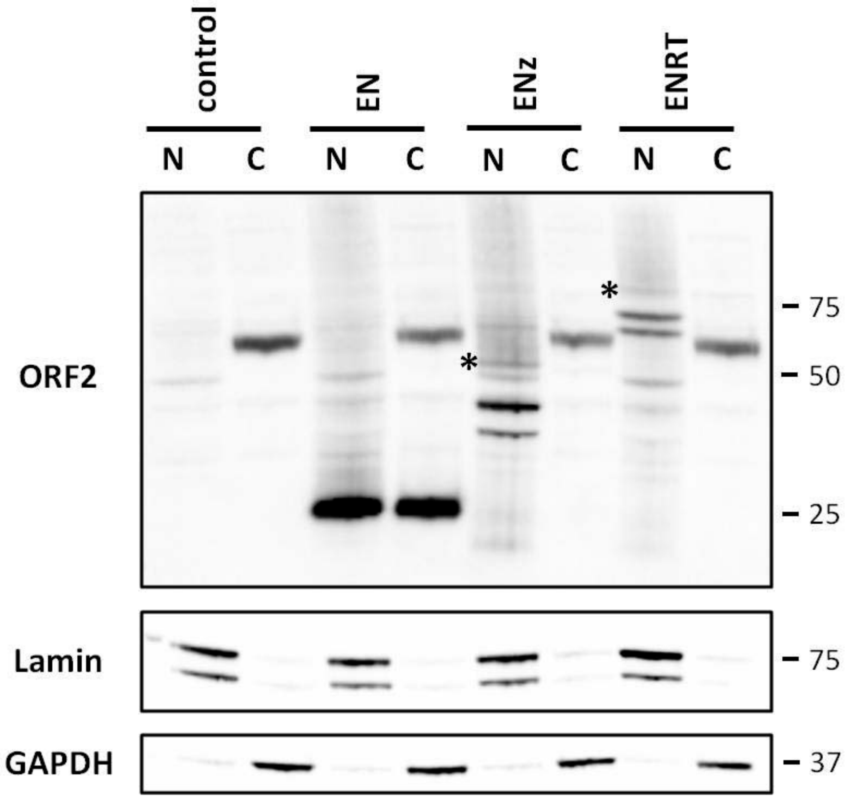

Supplementary Figure S8. Cellular localization of expressed truncated ORF2 proteins in HeLa cells. Representative western blot analysis of nuclear and cytoplasmic fractions from HeLa cells transiently transfected with truncated ORF2 expression plasmids. ORF2 panel, Truncated ORF2 proteins were detected with antibodies specific to the human L1 endonuclease domain; Lamin panel, anti-lamin antibodies were used as a cellular fractionation control (nuclear); GAPDH panel, anti-GAPDH antibodies were used as a cellular fractionation control (cytoplasmic). Asterisks indicate band of expected molecular weight. Control lanes indicate cells transfected with an empty vector. Molecular size markers are indicated on the right.

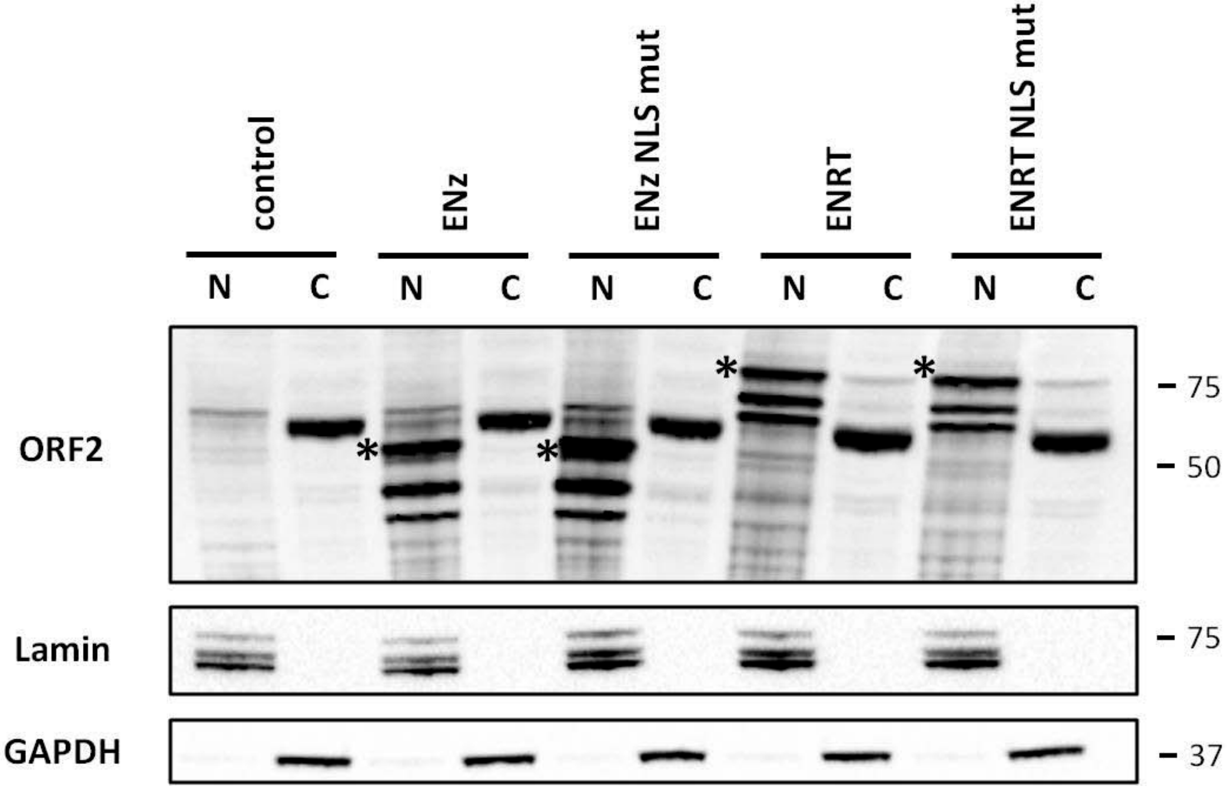

Supplementary Figure S9. Mutation of a putative nuclear localization signal in truncated ORF2 proteins. Representative western blot of nuclear and cytoplasmic fractions from 293 cells transiently transfected with truncated ORF2 or NLS mutant expression plasmids. ORF2 panel, Truncated ORF2 proteins were detected with antibodies specific to the human L1 endonuclease domain; Lamin panel, anti-lamin antibodies were used as a cellular fractionation control (nuclear); GAPDH panel, anti-GAPDH antibodies were used as a cellular fractionation control (cytoplasmic). Asterisks indicate band of expected molecular weight. Control lanes indicate cells transfected with an empty vector. Molecular size markers are indicated on the right.

L1 family	Location	Strand	Full-length	Size	L1 ORF- amino acid position of first stop codon	In a gene	% identity
L1PA4	chr1:165778070-165784153	-	yes	6084	ORF1 - 12	no	99.8%
L1PA6	chr1:220605521-220611692	-	yes	6172	ORF1 - 63	yes	99.4%
L1PA4	chr1:92629667-92635797	-	yes	6131	ORF1 - 34	yes	99.8%
L1PA4	chr2:149637881-149643991	-	yes	6111	ORF1 - 263	yes	98.5%
L1PA5	chr2:182686555-182692673	+	yes	6119	ORF1 - 42	no	98.0%
L1PA4	chr2:24800324-24806471	-	yes	6148	ORF1 - 77	yes	99.6%
L1PA6	chr3:136126021-136132173	+	yes	6153	ORF1 - 122	yes	99.9%
L1PA2	chr3:43565823-43571847	+	yes	6025	ORF1 - 109	yes	99.5%
L1PA3	chr3:67393030-67399181	+	yes	6152	ORF1 - 49	yes	99.8%
L1PA4	chr4:39246368-39252517	+	yes	6150	ORF1 - 93	yes	99.6%
L1PA5	chr5:36184264-36190366	-	yes	6103	ORF1 - 42	no	99.3%
L1PA3	chr5:79095900-79101910	-	yes	6011	ORF2 - 377	no*	99.8%
L1PA3	chr6:45176062-45181869	+	yes	5808	ORF1 - 41	yes	99.5%
L1PA3	chr7:128292012-128298140	+	yes	6129	ORF1 - 223	yes	99.1%
L1PA3	chr7:23321059-23327074	+	yes	6016	No identifiable start codon for ORF1.	yes	99.8%
L1PA4	chr7:77084514-77090684	-	yes	6171	ORF1 - 38	no	99.6%
L1PA5	chr9:21819141-21825252	-	yes	6112	ORF1 - 5	yes	99.5%
L1PA6	chr9:32957813-32963956	+	yes	6144	ORF1 - 42	no	99.5%
L1PA5	chr10:32912177-32918355	+	yes	6179	ORF1 - 15	yes	99.5%
L1PA6	chr11:14594761-14600876	-	yes	6116	ORF1 - 27	yes	99.1%
L1PA2	chr11:74877464-74883532	-	yes	6069	ORF1 - 190	yes	99.5%
L1PA5	chr12:100010760-100016815	+	yes	6056	ORF1 - 231	no	99.4%
L1PA6	chr12:45391884-45397988	+	yes	6105	No identifiable start codon for ORF1.	yes	99.9%
L1PA5	chr13:110669596-110675717	+	yes	6122	ORF1 - 63	yes	99.8%
L1PA4	chr14:88455734-88461846	-	yes	6113	ORF1 - 60	no	99.8%
L1PA3	chr15:43649976-43656002	+	yes	6027	ORF1 - 242	no	99.6%
L1PA5	chr18:70342942-70349081	-	yes	6140	ORF1 - 42	no	100.0%
L1PA4	chrX:70363246-70369397	-	yes	6152	ORF1 - 118	yes	99.6%

Supplementary Table S1. Genomic locations of full-length retrotranspositionally-*incompetent* L1 *loci* identified by RT-PCR analysis in HeLa cells. RT-PCR products were cloned, sequenced, and aligned to the human genome (GRCh38 assembly). The genomic location and characteristics of the L1 *loci* were recorded for unique clones. Transcripts corresponding to full-length L1 *loci* located within genes in the same orientation, could represent L1 sequences incorporated into cellular mRNAs and therefore not necessarily represent authentic L1 transcripts.

*****Recovery of an expressed transcript mapping to a full-length, retrotranspositionally-*incompetent* L1 *locus* with the potential to produce a truncated ORF2 protein is highlighted. This L1 *locus* is located outside of neighboring gene boundaries according to the UCSC Genes, RefSeq Genes, Genscan Gene, MGC Genes, ORFeome Clones, Pfam in UCSC Gene and UCSC Alt Events gene annotation tracks, and is predicted by ENCODE/GENCODE Genes V19 to be in a non-coding transcript specific to IFN-treated K562 cells (see Materials and Methods). A schematic is depicted in Supplementary Figure S1.
